# Proprioceptive Augmentation With Illusory Kinaesthetic Sensation in Stroke Patients Improves Movement Quality in an Active Upper Limb Reach-and-Point Task

**DOI:** 10.3389/fnbot.2021.610673

**Published:** 2021-03-01

**Authors:** Francesca Ferrari, Courtney E. Shell, Zachary C. Thumser, Francesco Clemente, Ela B. Plow, Christian Cipriani, Paul D. Marasco

**Affiliations:** ^1^The BioRobotics Institute, Scuola Superiore Sant'Anna, Pisa, Italy; ^2^Department of Excellence in Robotics & A.I., Scuola Superiore Sant'Anna, Pisa, Italy; ^3^Laboratory for Bionic Integration, Department of Biomedical Engineering, Lerner Research Institute-Cleveland Clinic, Cleveland, OH, United States; ^4^Advanced Platform Technology Center, Louis Stokes Cleveland VA Medical Center, Cleveland, OH, United States; ^5^Research Service, Louis Stokes Cleveland VA Medical Center, Cleveland, OH, United States; ^6^Department of Biomedical Engineering, Lerner Research Institute-Cleveland Clinic, Cleveland, OH, United States; ^7^Cleveland Clinic Lerner College of Medicine, Case Western Reserve University, Cleveland, OH, United States

**Keywords:** stroke, kinematics, reaching task, Fitts' law, sensory-motor rehabilitation, vibration illusion

## Abstract

Stroke patients often have difficulty completing motor tasks even after substantive rehabilitation. Poor recovery of motor function can often be linked to stroke-induced damage to motor pathways. However, stroke damage in pathways that impact effective integration of sensory feedback with motor control may represent an unappreciated obstacle to smooth motor coordination. In this study we investigated the effects of augmenting movement proprioception during a reaching task in six stroke patients as a proof of concept. We used a wearable neurorobotic proprioceptive feedback system to induce illusory kinaesthetic sensation by vibrating participants' upper arm muscles over active limb movements. Participants were instructed to extend their elbow to reach-and-point to targets of differing sizes at various distances, while illusion-inducing vibration (90 Hz), sham vibration (25 Hz), or no vibration was applied to the distal tendons of either their biceps brachii or their triceps brachii. To assess the impact of augmented kinaesthetic feedback on motor function we compared the results of vibrating the biceps or triceps during arm extension in the affected arm of stroke patients and able-bodied participants. We quantified performance across conditions and participants by tracking limb/hand kinematics with motion capture, and through Fitts' law analysis of reaching target acquisition. Kinematic analyses revealed that injecting 90 Hz illusory kinaesthetic sensation into the actively contracting (agonist) triceps muscle during reaching increased movement smoothness, movement directness, and elbow extension. Conversely, injecting 90 Hz illusory kinaesthetic sensation into the antagonistic biceps during reaching negatively impacted those same parameters. The Fitts' law analyses reflected similar effects with a trend toward increased throughput with triceps vibration during reaching. Across all analyses, able-bodied participants were largely unresponsive to illusory vibrational augmentation. These findings provide evidence that vibration-induced movement illusions delivered to the primary agonist muscle involved in active movement may be integrated into rehabilitative approaches to help promote functional motor recovery in stroke patients.

## Introduction

Stroke is one of the major causes of long-term disability (Plow et al., 2015). The effects of the stroke depend on several factors, including which brain regions are damaged. Whenever the affected regions include the motor and/or somatosensory cortex, motor, and sensory functions can be heavily compromised in the contralateral limb (Kitsos et al., 2013; Meyer et al., 2014). In recent years, an increasing number of rehabilitation therapies, including electrostimulation, repetitive task training, and robot-mediated therapies have been developed to recover impaired movement and lost function (Langhorne et al., 2009; Dipietro et al., 2012). Despite the wide range of motor rehabilitation therapies, 78% of patients never recover a normal level of motor performance (Potter-Baker et al., 2015).

In rehabilitation procedures, the desire to improve functional outcomes often biases therapy toward treatment of motor-related impairments, such as weakness, spasticity, and synergies, while omitting procedures that specifically integrate sensory feedback with motor control training (Bolognini et al., 2016). The functional limitations that persist after rehabilitation, despite normal muscle strength, suggest that insufficient or inappropriate restoration of sensory feedback may present a major obstacle to recovering smooth motor coordination (Bolognini et al., 2016).

Sensory impairments occur in 11–85% of patients post-stroke, affecting tactile sensation, stereognosis, and proprioception (Meyer et al., 2014). Proprioceptive feedback highly impacts motor recovery because it plays a key role in controlling muscle contraction by coordinating movements across multiple joints as well as promoting motor learning (Meyer et al., 2014; Findlater and Dukelow, 2017). Yet, the planning and execution of voluntary movements requires that the brain extract sensory information regarding body position and predict future positions by integrating a variety of sensory inputs with ongoing and planned motor activity (Scott, 2004). Various studies involving patients affected by sensory neuropathies have investigated the impact of proprioception in motor control. Although the lack of proprioceptive feedback does not prevent actual execution of a vast range of movements, it does impact movement quality (Rothwell et al., 1982; Ghez and Sainburg, 1995). In the absence of proprioception, movements are slow, clumsy, poorly coordinated, and inadequately adapted to complex tasks (Gordon et al., 1995); only supplementary integration of visual information can partially improve motor planning and control (Sainburg et al., 1995).

Proprioception is a complex sense. It arises from information provided by several different types of mechanoreceptors located in muscles, ligaments, joint capsules, and the skin (Riemann and Lephart, 2002). However, questions remain about the mechanism and functionality underpinning the integration of the proprioceptive sensors (Prochazka, 2011; Macefield and Knellwolf, 2018; Proske and Gandevia, 2018). Since 1972 (Goodwin et al., 1972a,b), superficial vibration applied over muscles or tendons has been used to investigate the role of proprioceptive neurophysiological components in providing information to the central nervous system (CNS) about limb movement and position (Roll and Vedel, 1982; Roll et al., 1989). These experiments demonstrated that vibration in the range of 80–100 Hz activates muscle receptors and elicits an illusion of movement associated with elongation of the vibrated muscle. In contrast, reducing the vibration frequency 10–30 Hz clearly diminishes the vividness of the illusions of movement as well as the response of the muscle receptors (Roll et al., 1989; Proske and Gandevia, 2018). The vibrations can generate movement illusions that are simple [e.g., elbow flexion-extension (Goodwin et al., 1972b)] or complex [e.g., writing-like movements (Roll and Gilhodes, 1995; Albert et al., 2006)].

The vibration-induced illusion of movement (i.e., kinaesthetic feedback) has been used in studies to restore sensory feedback (Cordo et al., 2013; Fusco et al., 2016; Marasco et al., 2018). In one of these studies, amputees who had undergone targeted reinnervation experienced complex illusions of movement (e.g., grasping movements) when a custom, wearable device (tactor) applied vibration to muscles that were part of their neural-machine interface (Marasco et al., 2018). These illusory movements matched the users' intended actions and the prosthesis moved in a way that matched both the intended action and the perceived movements (i.e., the action prediction was supported by receipt of the expected visual and proprioceptive feedback). The integration of the movement feedback provided by the vibration-induced illusion enabled amputees to precisely control the prosthesis movements at the level of able-bodied function in a grip preposition task (Marasco et al., 2018). Kinaesthetic feedback affected not only prosthesis control, but also promoted a sense of agency over prosthesis movements. Interestingly, contrary to perceived motions reported by able-bodied participants, vibration applied to muscles of targeted reinnervation amputees induced movement sensations corresponding to the action associated with muscle contraction, rather than extension. This observation suggests that muscle afferents activated by illusion-inducing vibration may signal active contraction rather than elongation.

There is limited evidence that the vibration-induced illusion of movement enhances proprioception after a stroke. Assistive robots have been paired with vibration-induced illusions of movement to promote the sensorimotor recovery of finger extension (Cordo et al., 2013). Motor functional improvements were tested with a box and block test at the end of treatments (2–3 months). Only less impaired participants (pre-therapy Upper Extremity Fugl-Meyer Assessment more than 17/66) showed improvements. During treatment, finger extension triggered vibration to the tendons of digit flexors and extensors combined with torque biofeedback or electromyographic (EMG) biofeedback, which was provided by horizontal bars on a screen indicating volitional torque or EMG. As the different feedback modalities were tested together, the specific contribution of kinaesthetic feedback to motor recovery was not evaluated.

In this study we investigate whether augmenting native kinaesthetic feedback with a matching vibration-induced movement illusion improves motor task performance of the affected upper limb of stroke patients. We test this hypothesis on patients with mild to moderate motor impairments and compare their task performance with that of able-bodied participants. In addition, by analyzing performance while the agonist or antagonist muscle received vibration-induced kinaesthetic feedback, we aim to verify how the kinaesthetic feedback, independent of participants' cognitive perception, provides relevant sensory information to the internal model.

Participants were instructed to perform a reaching task from a fixed starting position to a target that changed size and position with each trial (i.e., target width and distance from the starting position). Reaching tasks are often integrated in rehabilitation therapy (Thielman et al., 2004; McCrea and Eng, 2005). Furthermore, this type of task can be described and evaluated using Fitts' law (Fitts, 1954). Fitts' law quantifies the performance of rapid, aimed tasks as a relation between movement time and accuracy. Performance is expressed as a linear relationship between movement time and the log of the ratio between distance and width of the target. Fitts' law has been validated for both healthy participants (Wu et al., 2010; Sleimen-Malkoun et al., 2012; Thumser et al., 2018) and stroke patients (Thielman et al., 2004; McCrea and Eng, 2005; Kim et al., 2010). During the task, to restrict participants to using only proprioceptive information, arm movement was hidden from view. A motion tracking system was used to conduct a kinematic analysis of the arm trajectory.

To assess the effects of vibration-induced movement illusions on actual movement, both able-bodied participants and those who had experienced a stroke completed the reaching task with illusion-inducing (90 Hz), sham (25 Hz), and no vibration applied to the biceps and triceps brachii. In all cases, vibration was always applied only during the elbow extension. We expected that illusion-inducing vibration applied to biceps and triceps would cause participants to reach for the targets differently and that these responses would also be different than when no or sham vibration was applied. We also expected that the effects of vibration would be more evident for stroke patients than for able-bodied people.

## Materials and Methods

### Participants

Twelve naïve, able-bodied participants completed the reaching experiment with their dominant right arm (six female, 28 ± 9.3 years old, age range 19–48 years). All able-bodied participants had no deficits in mobility or sensation in either of their upper limbs. Six people who had experienced a stroke also participated in the study using their affected arm (Table 1; one female, three left-side affected, four ischemic, 57 ± 11.4 years old, age range 33–66 years). They were all in the chronic recovery phase, having had their stroke over a year prior to the experiment. All stroke participants were current or former participants of other larger clinical studies and were determined to have mild to moderate sensory-focused deficits by clinical study personnel through procedures and tests associated with those studies. Participants were referred specifically to our study by the clinicians coordinating other clinical studies in which they had previously participated and anticipated to be able to complete our tests (i.e., able to perform elbow flexion and extension). All participants were able to understand and carry out instructions provided in writing in the consent form as well as those given verbally by the experimenters. The Institutional Review Board of the Cleveland Clinic approved the study and all participants provided written informed consent prior to performing the experiment.

**Table 1:** Characteristics of stroke patient participants.

**Participant**	**Male/Female**	**Affected side**	**Years post-stroke**	**Age (years)**	Stroke subtype^**^	**Stroke lesion location**	**UEFM^†^ total**	**UEFM^‡^ hand and proximal**
S_01	M	Left	7.5	66	I	Pontomedullary Junction	65	29 and 36
S_02	M	Right	14.3	62	H	ø	ø	ø
S_03	F	Right	7.0	56	H	Left Thalamus	ø	ø
S_04	M	Right	3.8, 1.8^*^	66	I	Anterior Limb of the Internal Capsule, Putamen, Caudate	65	30 and 35
S_05	M	Left	5.8	61	I	Caudate, Putamen, Thalamus, Posterior Limb of Internal Capsule	59	27 and 32
S_06	M	Left	1.8	33	I	Brainstem	53	26 and 27

* *Note that S_04 had two ischemic strokes 2 years apart*.

** *Stroke Subtype is Ischemic (I) or Haemorrhagic (H)*.

† *Upper Extremity Fugl-Meyer Assessment (UEFM); total out of 66*.

‡ *Upper Extremity Fugl-Meyer Assessment (UEFM); total out of 30 (hand) and total out of 36 (proximal)*.

ø *Data unavailable*.

### Experimental Protocol

#### Determination of Location to Apply Vibration

**Figure 1:**
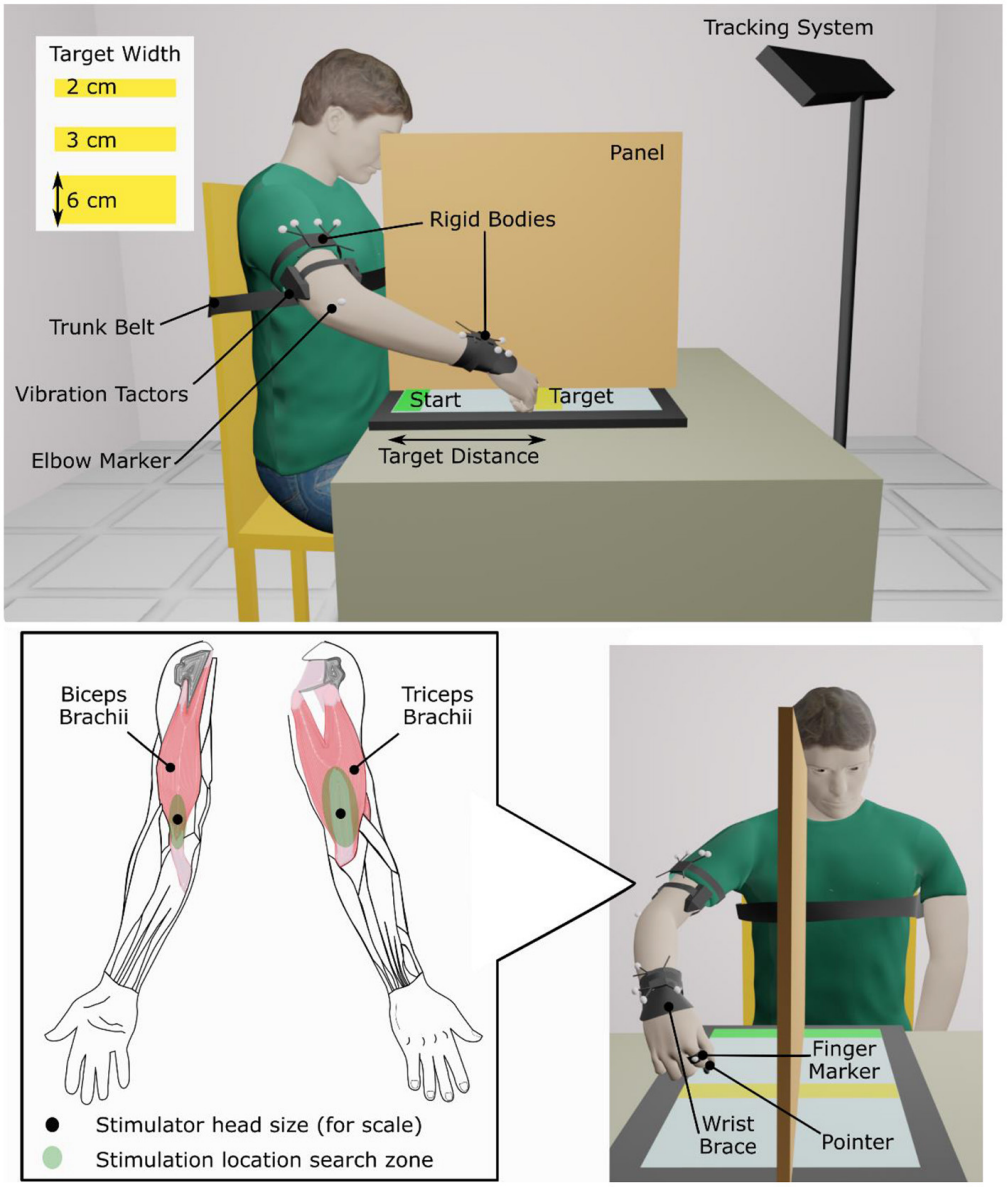
Experimental setup.

First, locations where 90 Hz, 1 mm-peak-to-trough vibration induced illusions of movement were found on the biceps brachii and the triceps brachii of the dominant arm for able-bodied participants and on the affected arm of people who had experienced a stroke. A hand-held vibration unit (Vibrasens VB200, TechnoConcept, Manosque, France) was placed at different points in a search zone on the distal part of the muscle around the myotendinous junction (Figure 1). Participants were asked to verbally report any movement sensation that they perceived. Experimenters encouraged participants to pay attention to sensations of movement about the elbow but gave no indication which direction the movement might take (i.e., flexion or extension). Locations where participants reported the strongest and most consistent movement about the elbow were selected and marked for later use. Some stroke patients did not report a clear elbow movement; in these cases, locations where patients reported a hint of movement of the forearm or shoulder were selected. A wearable vibration tactor (custom-made, HDT Global, Solon, OH, United States) was placed on each muscle over the identified locations.

#### Reaching Task

During the experiment, participants sat in an adjustable chair in front of a 77 × 47 cm touch screen laid flat on a table (Cintiq 27QHD, Wacom Co., Ltd., Kazo, Japan). A physical therapy gait belt secured participants' torsos to the back of the chair to prevent compensatory trunk movements during arm extension. Participants also wore a wrist brace that limited wrist flexion/extension and deviation (Figure 1).

Participants performed a reaching task during five different conditions: no vibration (NO VIB), illusion-inducing vibration applied to the biceps (BI 90), illusion-inducing vibration applied to the triceps (TRI 90), sham vibration applied to the biceps (BI 25), and sham vibration applied to the triceps (TRI 25). As in previous studies conducted by our research group (Marasco et al., 2017, 2018), these vibration frequencies were selected to compare the effects of a stimulus that clearly induces illusions of movement with simple vibration that does not induce movement illusions to serve as an additional control beyond the no vibration condition. In this task, participants moved from a starting position at the bottom of the screen to touch a target bar a random distance in front of them (Supplementary Video 1). First, the participant's maximum reach was determined by averaging four measurements of the farthest point on the screen that they could reach. A bar indicating the start position was constantly displayed at the near edge of the touch screen. Target bars were displayed one at a time, in a random order, at 20, 40, and 80% of the participant's maximum reach with widths of 2, 3, and 6 cm. Participants initiated each trial by touching the start bar, then reached and touched the target, then ended the trial by touching the start button again. To provide cues indicating when the trial was active, the start bar was pink before the first touch, became green when touched, stayed green during the trial, and then returned to pink when the second touch ended the trial. Target bars did not change appearance during the trial. A rubber-tipped pointer was fastened over the middle phalanx of participants' index or middle finger, according to individual preference. Participants kept their hand closed and touched the screen with the pointer only. They were instructed to complete each reach from the start bar to the target bar as quickly and accurately as possible using one sustained movement. During the experiment, participants received no information regarding trial performance or whether they touched the target or not.

One block of trials consisted of one presentation of each target, requiring nine reaches per block. Participants practiced the task with vision and no vibration (at least one block). Then, a vertical panel was arranged in the middle of the touch screen, hiding from view the arm that performed the reaching task. Target and start bars extended across both halves of the screen so that they remained visible. Participants practiced the task without visual feedback of arm movement and with no vibration (at least two blocks) until comfortable. After three to five total practice blocks and with the vertical panel in place, three experimental blocks were collected for each condition (5 vibration conditions ×3 blocks of 3 target distances ×3 target widths = 135 trials total).

A marker-based motion capture system tracked arm movements (Optitrack V120:Trio, NaturalPoint, Inc., Corvallis, OR, United States). Two clusters of four markers, each, were fastened on the arm, one above the elbow and the other on the forearm just above the wrist. Two additional markers were applied, one on the proximal phalanx of the finger to which the pointer was attached and the other on the lateral epicondyle of the elbow. These markers tracked the pointer location (and thus the hand) and aided calculations of elbow angle, respectively. In conditions where vibration was applied, tactor activation was synchronized with elbow extension so that the appropriate muscle received 90 Hz, movement-illusion-inducing vibration or slower frequency (25 Hz) sham vibration as a control condition.

To run the experiment and collect data, two custom applications (LabVIEW, National Instruments, Austin, TX, United States) were run in parallel on two computers. The first computer, connected to the touch screen, controlled the display and recorded the positions and times the pointer contacted the screen. The second computer, connected to the motion capture system and to the two vibration tactors, controlled vibration tactor activation and recorded marker and cluster centroid positions as well as vibration duration. A trigger indicating the start and stop of each trial was sent between computers to disable the tactors between one trial and the next as well as to synchronize data during post-processing analysis.

At the end of each experimental block, participants were asked to verbally answer two questions with a rating on a scale from 0 to 100. These two questions gauged participants' self-perceived accuracy (“How confident are you that you accurately touched the target buttons where 0 means ‘not confident at all’ and 100 means ‘absolutely sure/confident’?”) and fatigue (“What was your level of fatigue during the last experimental block where 0 is ‘no fatigue’ and 100 is ‘greatest possible fatigue’?”).

### Data Analysis and Variable Definition

All data were aggregated across all participants of each group, able-bodied and stroke patients, and they were processed with a custom MATLAB script (R2011a, Mathworks, Natick, MA, United States) and analyzed using SPSS (IBM Corp., United States). Arm kinematics and Fitts' parameters were examined for the movement from the start to the target position, excluding the return movement from the target back to the start position. In addition, subjective ratings of performance accuracy and fatigue were evaluated.

#### Kinematics

Kinematics were analyzed for the elbow angle and the marker over the finger to which the pointer was attached. Marker data were smoothed with a 4th order, lowpass Butterworth filter with a cut-off frequency of 7 Hz. The cut-off frequency was determined through residual analysis of the zero derivative by regression (Mullineaux, 2017). All trials with markers missing for more than 200 ms or for more than 10% of the whole trial duration were eliminated.

Directness and smoothness of the hand marker movement were calculated to characterize the movement trajectories. Directness was calculated as the ratio between the length of the actual trajectory and the length of an ideal straight path connecting the initial and the final position (Bastian et al., 1996; McCrea and Eng, 2005). A separate analysis was also conducted to quantify deviations of the trajectory from a linear path in the sagittal and transverse planes. Smoothness was quantified by the number of peaks in the magnitude of the three-dimensional velocity divided by the length of the trajectory from the initial to the final position (Gulde and Hermsdörfer, 2018).

In order to compare arm extension across conditions, the elbow angle, defined with the vertex located at the single marker over the elbow epicondyle with rays extending to the centroids of the marker clusters on the upper arm and the forearm, was calculated using the cosine rule. The elbow angle was normalized and expressed as a percentage of the elbow angle in the NO VIB condition [e.g., (Elbow Angle _BI 90_ − Mean(Elbow Angle _NO VIB_))/Mean(Elbow Angle _NO VIB_)^*^100] to account for different maximum distances reached by each participant.

Finally, the velocity profile of the pointer finger trajectory was analyzed. The peak velocity (PV) was defined as the maximum velocity reached between 10 and 100% of the movement and the time to peak velocity (TTP) was the time necessary to reach the peak velocity measured from the beginning of the trial.

#### Fitts' Parameters

Movement performance across the different experimental conditions was characterized by applying Fitts' law. Fitts' law relates the duration of the movement, distance, and pointing accuracy to describe movement performance. We calculated the effective index of difficulty (IDe) using the Shannon formulation with accuracy adjusted for each participant, target, and experimental condition using the following equation (Soukoreff and MacKenzie, 2004),

$$\begin{array}{l} IDe=\log_{2\;}\left(\frac{De}{We}+1\right). \end{array}$$

Effective distance (De) was defined for each target as the mean movement distance from the initial position of the movement (actual touched point within the start button) to the end position (first touched point within the second half of the distance from the starting position to the prescribed target) across the three repetitions. Effective target width (We) was calculated for each target in each experimental condition as

$$\begin{array}{l} We=\sqrt{2\pi e\sigma^{2}}=4.133\sigma , \end{array}$$

where σ indicates the standard deviation of the end position in the direction of motion (first touched point closer to the target) across the three repetitions. *We* therefore described the target region each participant touched 96% of the time (Soukoreff and MacKenzie, 2004). The ratio between IDe (measured in bits of information) and the movement time (MT) defined the throughput (TP) index, which evaluates the movement performance in terms of both speed and accuracy. TP (expressed in bit/s) was calculated for every condition for each participant as the mean TP of all of the presented targets, T (i.e., all target distance and width combinations), and then averaged across the number of participants (N),

$$\begin{array}{l} TP=\frac{1}{N}\sum_{i=1}^{N}\left(\frac{1}{T}\;\sum_{j=1}^{T}\frac{IDe_{ij}}{MT_{ij}}\;\right). \end{array}$$

The formula used for calculating IDe was also used to obtain the prescribed, as opposed to effective, index of difficulty (ID) using the actual distance (i.e., distance from the bottom of the starting position to the middle of the target) and target width (Soukoreff and MacKenzie, 2004). Note that participants were not presented with targets of identical prescribed IDs because target distances were adjusted according to the furthest point each participant could reach. Thus, additional analyses were also performed to separately evaluate the ratio between the effective and the prescribed distance (De/D) and the normalized movement time. Normalized movement time was expressed as a percentage of the participant's average movement time in the NO VIB condition [e.g, (MT_BI 25_ − Mean(MT_NO VIB_))/Mean(MT_NO VIB_)^*^100, where Mean(MT_NO VIB_) was calculated for each target considering the three repeated reaches of the NO VIB condition].

#### Questionnaire

For each participant, the subjective ratings of fatigue and self-perceived movement accuracy were averaged across the three repetitions of each experimental condition. Then, the average ratings for each condition were compared across participants.

### Statistical Analysis

Comparisons were made between experimental conditions by fitting linear mixed models to individual measures. Linear mixed models are well-suited to analyzing repeated measures data, are efficient for small sample sizes, and are robust to instances of missing data (West, 2009; Muth et al., 2016). This analysis technique allowed measures from individual trials to be included, rather than an average across repetitions for each participant, and maximized the statistical power of the analysis. Models included fixed effects for vibration condition (five levels), target distance (three levels), and an interaction effect between vibration condition and target distance. A subject-specific random intercept was included to account for differences between participants. Heterogeneous participant variance and no correlation between participants were assumed (diagonal covariance structure). To account for the repetitions of each condition, a repeated factor was introduced with equal trial variance and no correlation between trials (scaled identity covariance structure). In graphical representations of the data, we show results using box plot graphs, which indicate the data distribution between the 25th (Q25) and the 75th (Q75) percentile. Outliers were considered those values larger than [Q75 + 1.5(Q75 − Q25)] or lower than [Q25 − 1.5(Q75 − Q25)] and the whiskers shown encompass all non-outlier values. No outliers were removed for data analysis or determination of significant differences. Significant main and interaction effects were investigated using Bonferroni-corrected *post-hoc* tests (α = 0.05). A similar analysis was conducted for the IDe. However, because the calculation of IDe already included the standard deviation (from the calculation of effective width, We), the repetition effect was excluded from this analysis. The throughput, TP, and questionnaire responses on fatigue and accuracy were separately evaluated using one-way repeated measures ANOVAs (RM-ANOVA) and Bonferroni-corrected pairwise analyses (α = 0.05).

## Results

Participants were instructed to perform a reaching task from a fixed starting position to a target that changed size and position with each trial (i.e., target width and distance from the starting position). Results are reported for the analysis of the arm kinematics, the Fitts' parameters, and the questionnaire. Regarding the kinematic analysis, 0.3 and 1.6% of the trials were eliminated, respectively for the able-bodied and the stroke patient participants because of missing markers in the motion tracking data.

### Kinematics

#### Directness

**Figure 2:**
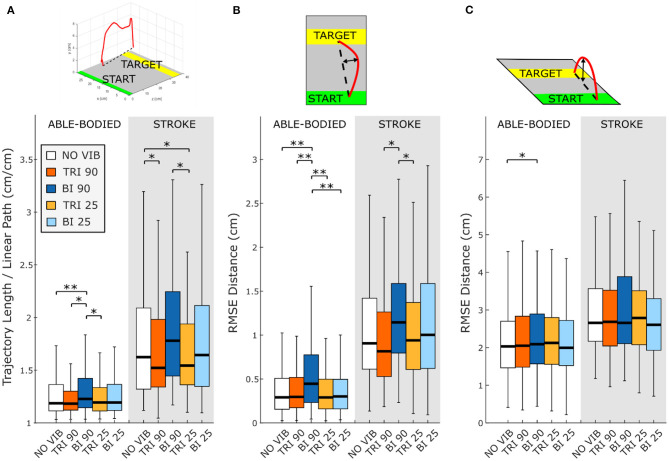
Directness is measured by **(A)** the ratio between the length of the traveled trajectory and the length of a linear path between the starting and the final positions as well as the root mean square errors (RMSE) of the distance of the trajectory from the linear path projected in the **(B)** transverse plane and **(C)** sagittal plane. Higher values indicate a less direct trajectory. In each subplot medians and interquartile ranges [25th and 75th percentile (Q25, Q75)] with whiskers indicating the range of non-outlier values are shown for data aggregated across all participants and targets for both able-bodied and stroke patient participants when no vibration (white, NO VIB), 90 Hz vibration on the triceps (orange, TRI 90), 90 Hz vibration on the biceps (blue, BI 90), 25 Hz vibration on the triceps (yellow, TRI 25), and 25 Hz vibration on the biceps (light blue, BI 25) was applied. The statistical differences indicated (^*^p < 0.05, ^**^p < 0.001) refer to the main effect of the experimental condition.

The directness of the movement trajectory (i.e., trajectory length/linear path length) revealed a difference between vibration on the biceps and the triceps (main effect, *p* < 0.001, Figure 2A). Able-bodied participants showed a significantly less direct trajectory when the biceps was vibrated at 90 Hz (compared to no vibration *p* < 0.001, triceps vibrated at 90 Hz *p* = 0.001, and triceps vibrated at 25 Hz *p* = 0.003). Similar, but not all significant, decreases in directness were observed in stroke patients (compared to triceps vibrated at 90 Hz *p* = 0.052 and triceps vibrated at 25 Hz *p* = 0.026). Conversely, stroke patients moved in a more direct path when vibration was applied to the triceps (comparing no vibration with triceps vibrated at 90 Hz *p* = 0.006, and triceps vibrated at 25 Hz *p* = 0.003). A significant main effect of target distance (*p* < 0.001) as well as a significant interaction effect between vibration condition and target distance (*p* < 0.050) was also found for both participant groups. Significant differences between vibration conditions were present only for targets at 20% of the maximum reachable distance although similar but non-significant differences were found for the targets presented at 40 and 80% of the maximum reachable distance (Supplementary Figure 1).

**Figure 3:**
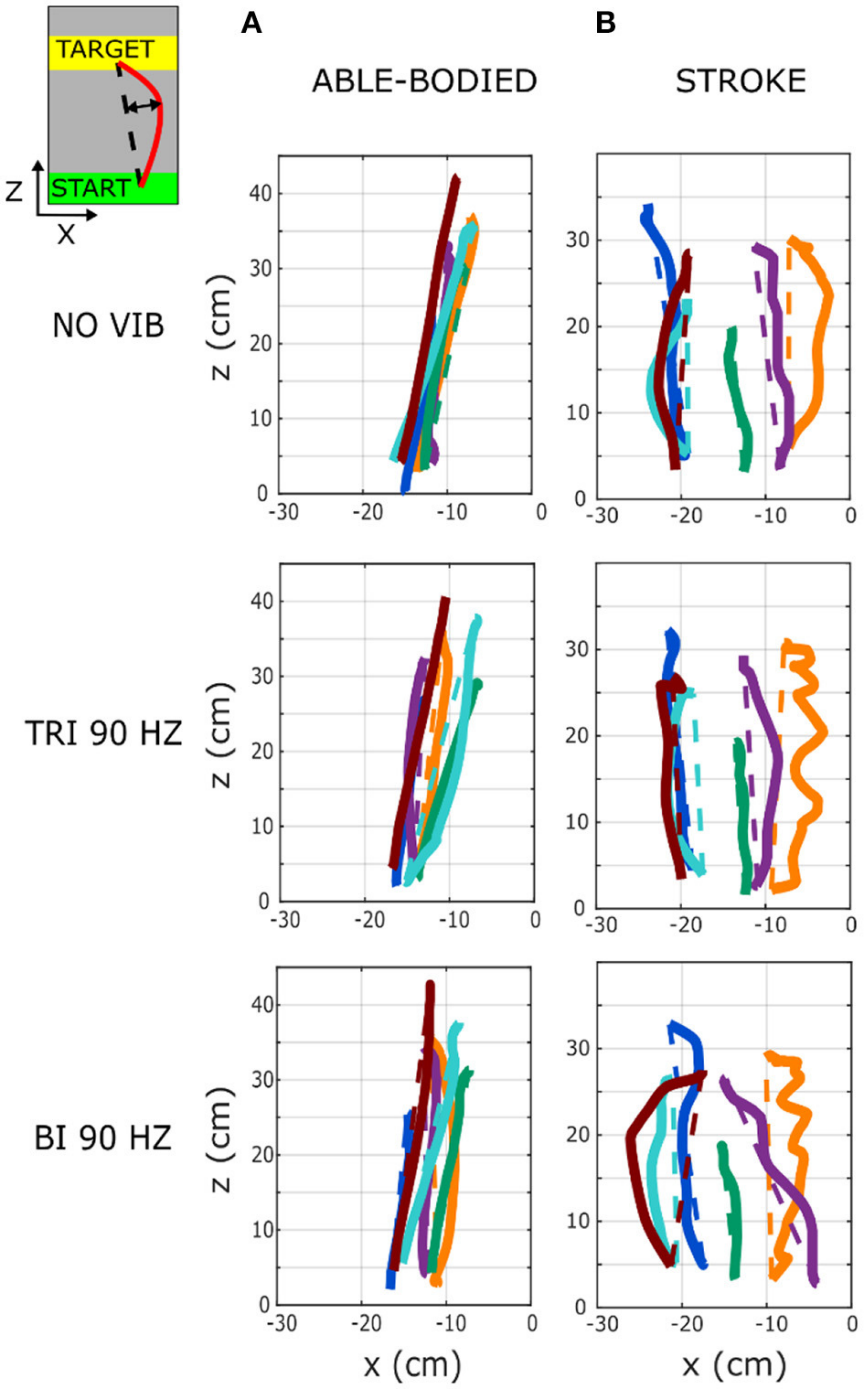
Six representative movement trajectories projected in the transverse plane, each from a different **(A)** able-bodied and **(B)** stroke patient participant reaching a target set at 80% of the maximum reachable distance during three experimental conditions: no vibration (NO VIB), 90 Hz vibration applied to the triceps (TRI 90), and 90 Hz vibration applied to the biceps (BI 90). Dashed lines represent the ideal linear path while the solid lines represent the actual trajectory.

A separate analysis of divergence from the linear path in the sagittal and the transverse planes showed that most deviations occurred in the transverse plane (Figures 2B,C). Greater divergence indicates more curves and directional changes in the trajectory (Figure 3). The vibration condition and target distance affected divergence in the transverse plane in both able-bodied and stroke patient participants (target distance *p* < 0.001 for both participant groups, vibration condition *p* < 0.001 for able-bodied and *p* < 0.050 for stroke patients). The deviation increased for both participant groups, especially when 90 Hz vibration was applied to the biceps [able bodied: comparing 90 Hz vibration on the biceps to no vibration *p* = <0.001, triceps vibrated at 90 Hz *p* < 0.001, triceps vibrated at 25 Hz *p* < 0.001, and biceps vibrated at 25 Hz *p* < 0.001; stroke patients comparing the biceps vibrated at 90 Hz to triceps vibrated at 90 Hz *p* = 0.002, triceps vibrated at 25 Hz *p* = 0.046, no vibration *p* = 0.383 and biceps vibrated at 25 Hz (*p* ≥ 1); Figure 2B]. There was also a significant interaction effect between vibration condition and target distance for able-bodied participants (*p* = 0.011, Supplementary Figure 2A). Compared to no vibration, 90 Hz vibration applied to the biceps caused significantly greater divergence from the linear path across all target distances (at 80% *p* < 0.001, 40% *p* = 0.008, and 20% *p* = 0.014 of the maximum reachable distance). Fewer significant differences occurred in the sagittal plane. Only able-bodied participants showed any significant differences, with main effects of both target distance (*p* < 0.001) and vibration condition (*p* = 0.008) as well as an interaction effect (*p* = 0.003). The interaction effect revealed significant differences only for targets at 80% of the maximum reachable distance, where 90 Hz vibration of the triceps caused significantly greater divergence than all experimental conditions (comparing 90 Hz vibration on the triceps to no vibration *p* < 0.001, biceps vibrated at 25 Hz *p* = 0.016, triceps vibrated at 25 Hz *p* = 0.009) except 90 Hz vibration of the biceps (*p* = 0.801) (Supplementary Figure 2B). Therefore, application of 90 Hz vibration to the triceps caused a slightly higher hand movement trajectory with respect to the screen. No significant differences were observed in the movement trajectories in the sagittal plane for the stroke patients (Figure 2C).

#### Smoothness

**Figure 4:**
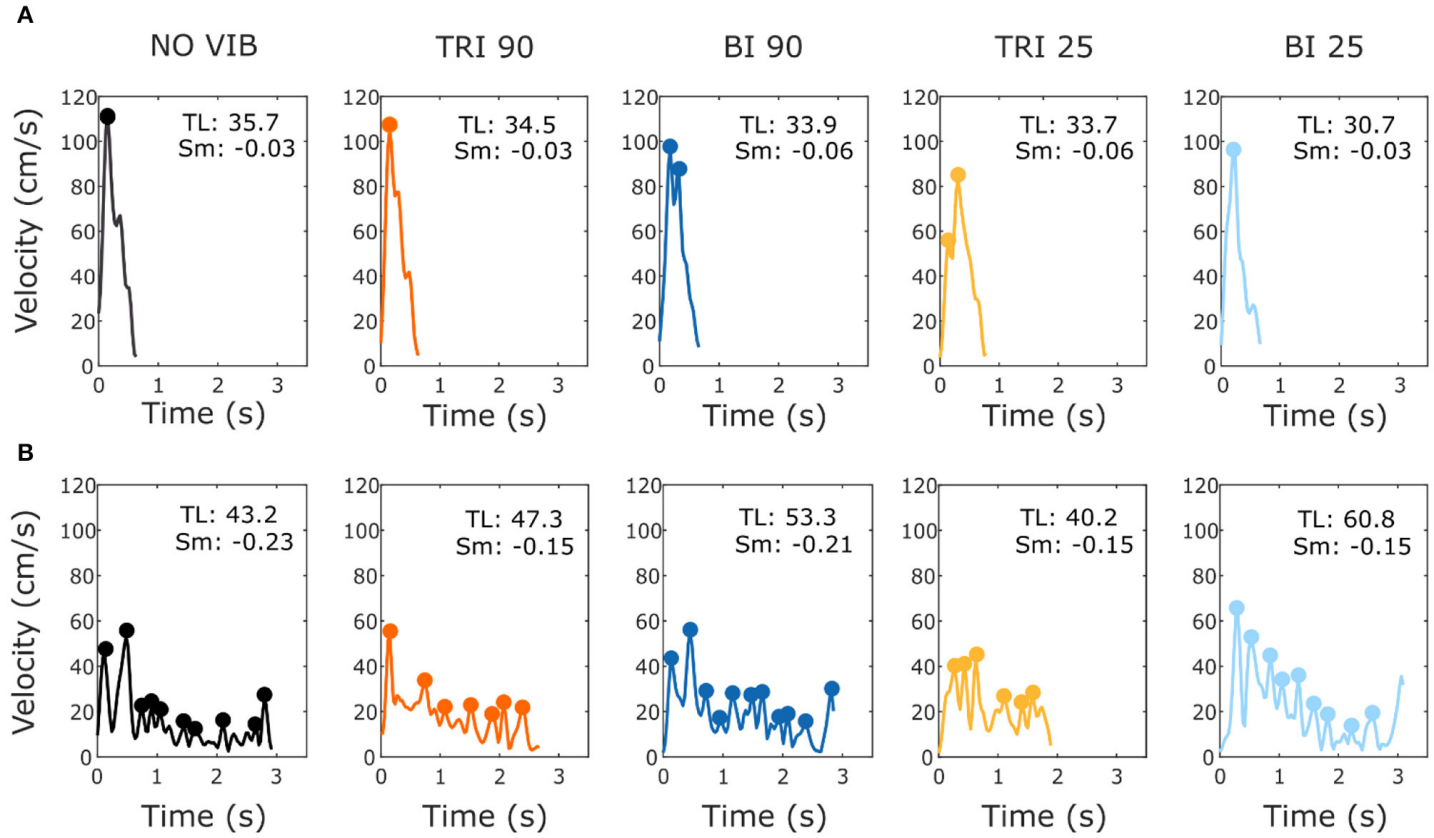
Example velocity profiles from **(A)** one able-bodied participant and **(B)** one stroke patient for the five experimental conditions while reaching a target at 80% of the maximum reachable distance. The dots represent the local peaks with a minimum prominence of 5 cm/s. The numbers in the right corner of each graph represent the traveled trajectory length (TL) and the smoothness index (Sm: -n. peaks/trajectory length). Higher (less negative) smoothness index values indicate a smoother movement.

**Figure 5:**
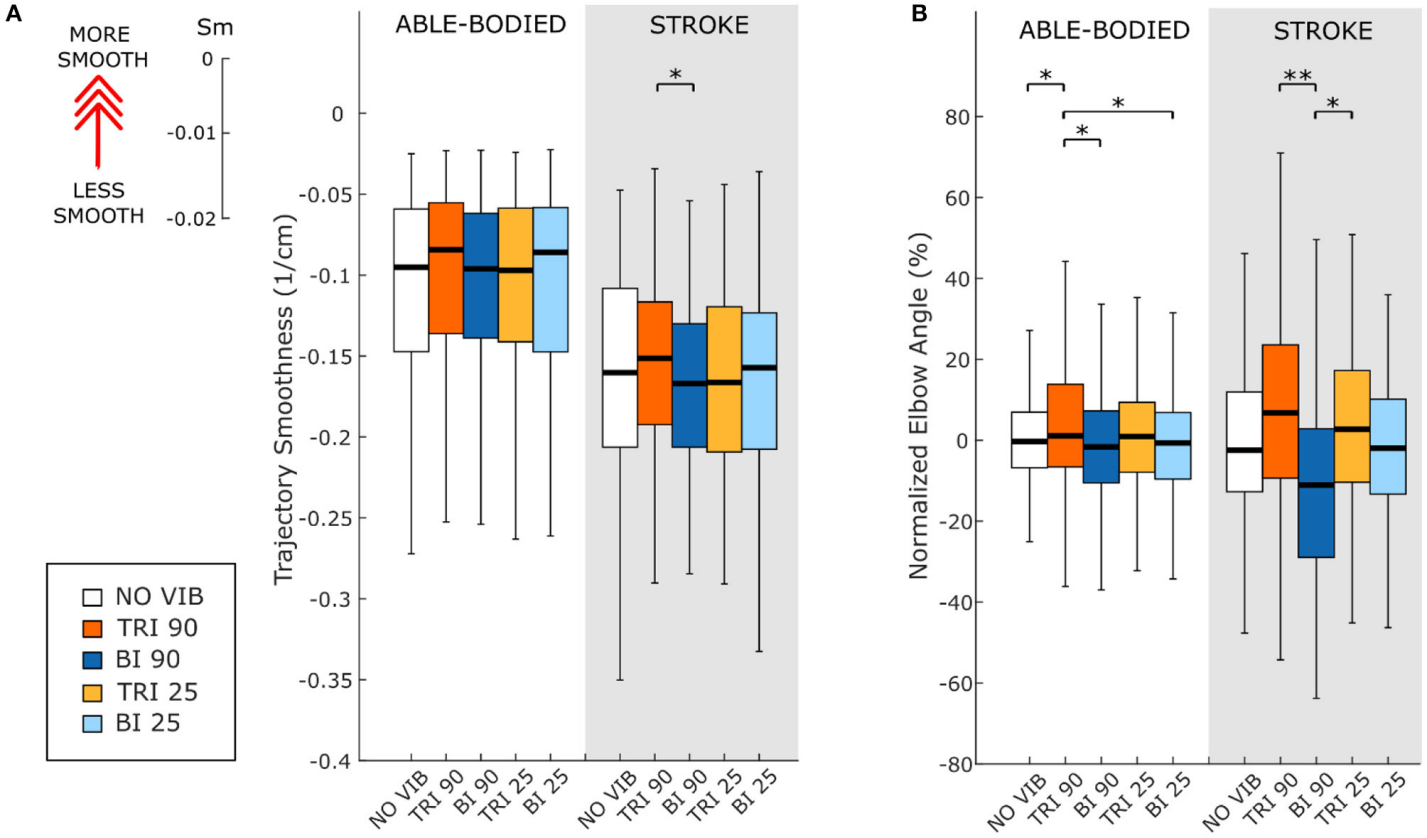
**(A)** Movement smoothness was quantified as [-(number of velocity peaks/trajectory length)], where higher (less negative) values represent smoother movements. **(B)** Peak elbow angles were normalized and averaged across 12/12 able-bodied and 5/6 stroke patient participants. In each subplot medians and interquartile ranges [25th and 75th percentile (Q25, Q75)] with whiskers indicating the range of non-outlier values are shown for data aggregated across all participants and targets for both able-bodied and stroke patient participants when no vibration (white, NO VIB), 90 Hz vibration on the triceps (orange, TRI 90), 90 Hz vibration on the biceps (blue, BI 90), 25 Hz vibration on the triceps (yellow, TRI 25), and 25 Hz vibration on the biceps (light blue, BI 25) was applied. The statistical differences indicated (^*^p < 0.05, ^**^p < 0.001) refer to the main effect of the experimental condition.

As expected, the able-bodied participants moved more smoothly than the stroke patients and mostly show a bell-shaped velocity profile with a single peak (Figure 4A). No significant differences were found between the movement smoothness in the five experimental conditions for the able-bodied participants, where only the target distance had a significant effect (*p* < 0.001) (Figure 5A). In contrast, stroke patients moved less smoothly with a greater number of changes in velocity (Figure 4B). Both target distance (*p* < 0.001) and the experimental condition (*p* < 0.050) had significant main effects, with 90 Hz vibration of the triceps leading to a significantly smoother trajectory than 90 Hz vibration of the biceps (*p* = 0.007) (Figure 5A).

#### Movement Extension

The effect of vibration condition on the elbow angle aligns with the effects on Fitts' law measures of distance (De/D). Larger elbow angles were attained (i.e., reached farther) by both able-bodied and stroke patient participants when the triceps was stimulated (Table 2). However, data from only five of the six stroke patients were included because there were technical problems tracking upper arm markers in one of the participants. The target distance and the vibration condition had a main effect on the elbow angle (main effect of the target distance for both participant groups *p* < 0.001; main effect of the vibration condition *p* < 0.050 and *p* < 0.001 for the able-bodied participant and the stroke patients, respectively). Vibration on the triceps at 90 Hz caused significantly greater elbow angles in both participant groups (able-bodied: compared to biceps vibrated at 90 Hz *p* = 0.007; stroke: compared to biceps vibrated at 90 Hz *p* < 0.001 and biceps vibrated at 25 Hz *p* = 0.035, Table 2). Normalized elbow angles (Figure 5B) were similarly affected (*p* < 0.050 main effect of the target distance only for able-bodied, and *p* < 0.050 and *p* < 0.001 for the experimental condition for the able-bodied and patients, respectively). In particular, when compared to the no vibration condition, 90 Hz vibration applied on the triceps led to a 6.5% (stroke patients) and 1% (able-bodied) larger elbow angle. On the other hand, when 90 Hz vibration was applied to the biceps, the elbow angle decreased 1.5% (able-bodied) and 11% (stroke patients).

**Table 2:** The median, 25th percentile and the 75th percentile values of the elbow angle expressed in degrees for each experimental condition for both able-bodied (AB) and stroke patient (PAT) participants.

	**NO VIB**		**TRI 90**		**BI 90**		**TRI 25**		**BI 25**	
**Elbow angle (°)**	**AB**	**PAT**	**AB**	**PAT**	**AB**	**PAT**	**AB**	**PAT**	**AB**	**PAT**
Median	22.9	19.9^b^	23.4^b^	21.9^bb^	23.2^t^	16.4^tt^	23.8	19.8^bb^	23.7	19.7^b, t^
25th Percentile	10.4	10.9	10.5	11.6	9.6	9.1	10.1	10.2	10.4	9.9
75th Percentile	52.4	35.3	52.1	39.9	47.7	30.7	49.5	37.1	48.4	30.5

Statistically significant differences in the main effect of the experimental condition are reported compared to the biceps vibrated at 90 Hz (

b p < 0.05 and

bb p < 0.01)

and compared to the triceps vibrated at 90 Hz (

t p < 0.05 and

tt *p < 0.001)*.

#### Velocity and Time to Peak Velocity

Vibration condition did not significantly affect the velocity and the time to velocity peak (velocity: *p* = 0.882 for able-bodied and *p* = 0.120 for stroke; time to velocity peak: *p* = 0.717 for able-bodied and *p* = 0.637 for stroke). There was a significant main effect of target distance (*p* < 0.001 for both groups, Table 3) but no significant interaction effects (velocity: *p* = 0.808 for able-bodied and *p* = 0.364 for stroke; time to velocity peak: *p* = 0.729 for able-bodied and *p* = 0.084 for stroke). Similar results were found for the normalized time defined as the percentage of the time to peak with respect to the duration of the entire movement (main effect of target distance *p* < 0.001, Table 3).

**Table 3:** Mean value of the velocity, time to velocity peak and normalized time to peak for the three different target distances for all the participants across all experimental conditions.

	**20% maximum reach**		**40% maximum reach**		**80% maximum reach**	
**Mean and standard deviation**	**AB**	**PAT**	**AB**	**PAT**	**AB**	**PAT**
Velocity peak (cm/s)	34.1 ± 10.7	35.3 ± 12.3	57.3 ± 13.7	40.9 ± 10.2	96.7 ± 21.0	51.7 ± 12.3
Time to peak (s)	0.19 ± 0.15	0.47 ± 0.36	0.20 ± 0.14	0.46 ± 0.34	0.24 ± 0.13	0.59 ± 0.45
Time to peak/Total trial time (%)	43 ± 24	59 ± 31	38 ± 18	52 ± 29	35 ± 13	44 ± 25

*PAT, stroke patients; AB, able-bodied*.

### Fitts' Law Parameters

**Figure 6:**
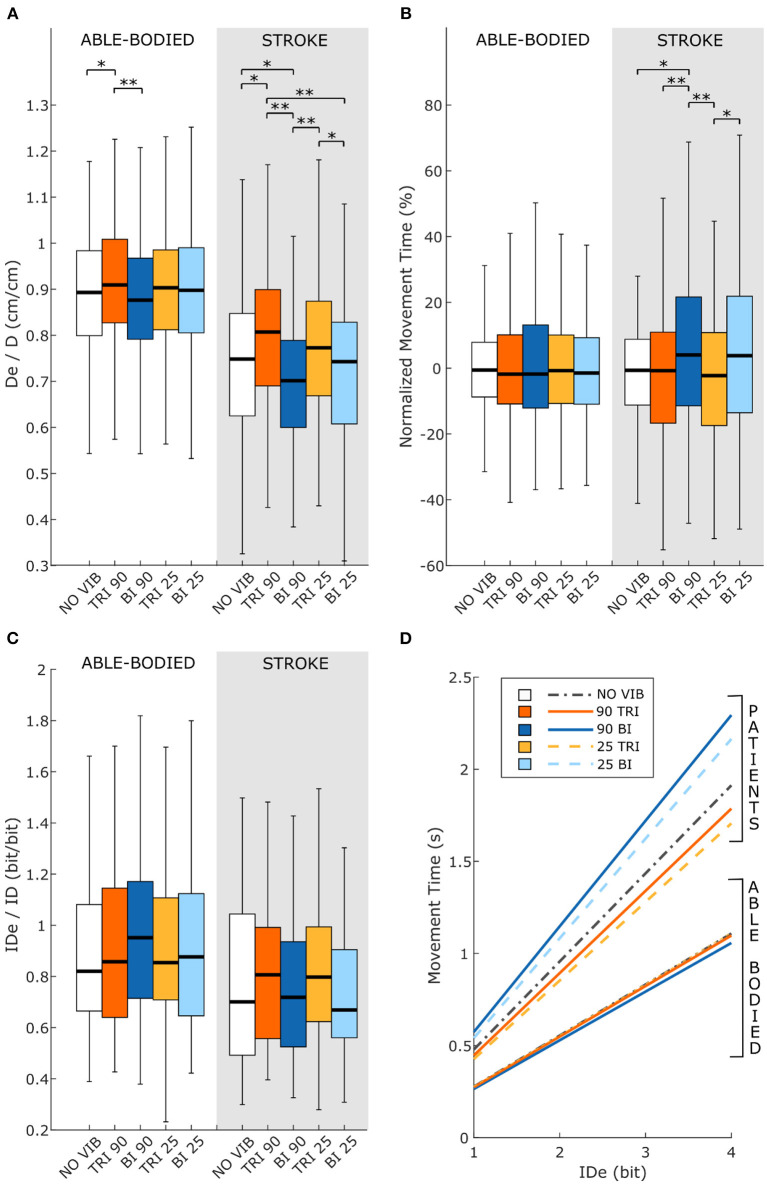
Fitts' law parameters included **(A)** the expected over the prescribed target distance (De/D), **(B)** the normalized movement time, and **(C)** the ratio between the effective and prescribed index of difficulty (IDe/ID). In each subplot medians and interquartile ranges [25th and 75th percentile (Q25, Q75)] with whiskers indicating the range of non-outlier values are shown for data aggregated across all participants and targets for both able-bodied and stroke patient participants. The statistical differences indicated (^*^p < 0.05, ^**^p < 0.001) refer to the main effect of the experimental condition. **(D)** The relationship between the movement time and the effective index of difficulty averaged across all participants in the two groups, stroke patients and able-bodied participants, is also shown. The slopes of the lines in **(D)** represent the inverse of the throughput, which is expressed in bit/s.

Statistical analysis of the throughput (TP) did not reveal any significant differences between the experimental conditions for either group of participants [RM-ANOVA, *F*_(4, 44)_ = 0.27 *p* = 0.899 for able-bodied, and RM-ANOVA, *F*_(4, 20)_ = 1.78, *p* = 0.172 for stroke patients]. Stroke patients tended to show a slightly higher TP when the triceps brachii was vibrated compared to when the biceps brachii was vibrated (Figure 6D). This indicates that, with the same level of target difficulty (i.e., IDe), stroke patient participants completed the task in a shorter time. To explore the source of changes in TP, a separate analysis was conducted on the individual Fitts' law parameters (De/D, normalized MT, and IDe/ID).

Vibration condition affected the De/D measure in both able-bodied and stroke patient participants (main effect, *p* < 0.001, Figure 6A). For both groups, 90 Hz vibration on the triceps increased De/D, shifting it closer to 1, compared to 90 Hz vibration on the biceps (*p* < 0.001) or no vibration (*p* < 0.050). The shift in De/D closer to 1 indicates that stimulating the triceps brachii allowed the participants to better approach the target distance. A significant interaction effect between the vibration condition and the target distance was observed only for the stroke patients (*p* = 0.309 for able-bodied and *p* = 0.034 for stroke patients). This interaction effect revealed that the results were more evident for targets at 20 and 40% of the maximum reachable distance (Supplementary Figure 3A).

The IDe/ID did not change significantly between conditions (*p* = 0.759 for able-bodied and *p* = 0.228 for stroke patients) nor was there a significant interaction effect between target distance and experimental condition for both able-bodied and stroke patients (*p* = 0.790 for able-bodied and *p* = 0.305 for stroke patients) (Figure 6C).

The analysis of the normalized MT revealed that the vibration condition caused differences only in stroke patients (*p* = 0.134 for able-bodied; *p* < 0.001 for stroke patients), who took longer to reach the targets when the biceps received 90 Hz vibration compared to no vibration (median value 4% higher, *p* = 0.009) or vibration on the triceps (median 4% higher than 90 Hz vibration, *p* < 0.001, and 5% higher than 25 Hz vibration, *p* < 0.001) (Figure 6B). There was also a significant interaction effect between the target distance and the vibration condition (*p* = 0.047). *Post-hoc* analyses of the interaction effect suggested that differences between vibration conditions were significant only for targets at 80% of the maximum reachable distance (Supplementary Figure 3B). For this group of targets farthest away from the participants, 90 Hz vibration on the biceps caused longer movement times, around 15% more (median value) than the no vibration condition and significantly different than all other conditions (biceps vibrated at 90 Hz compared to biceps vibrated at 25 Hz *p* = 0.049, compared to all other conditions *p* < 0.001). The other vibration conditions caused normalized movement time percentages to oscillate between 0 and 4% of the average movement time when no vibration was applied, and none were significantly different from the no vibration condition (comparing no vibration to biceps vibrated at 25 Hz *p* = 0.366 and both triceps vibrated at 90 Hz and triceps vibrated at 25 Hz *p* ≥ 1.000).

### Questionnaires

**Figure 7:**
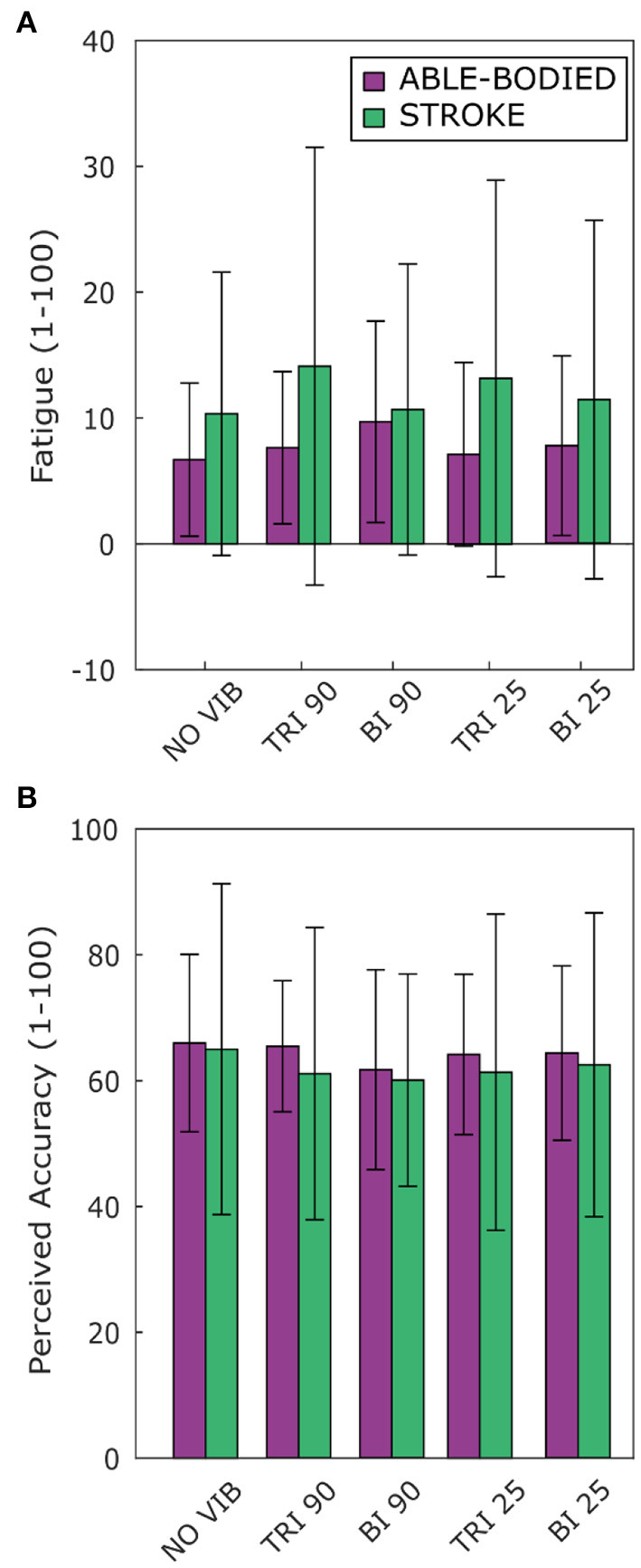
Questionnaire results for perceived **(A)** fatigue and **(B)** movement accuracy during the task reported by both able-bodied (purple) and stroke patient participants (green). The mean value and the standard deviations were calculated across the ratings of the three repetitions of each experimental condition.

Vibration condition affected neither perceived fatigue nor self-reported movement accuracy. No statistical differences were found between conditions for both able-bodied and stroke patient participants [Fatigue: RM-ANOVA, *F*_(2.12, 23.34)_ = 2.02, *p* = 0.107 for able-bodied, and RM-ANOVA, *F*_(1.15, 5.75)_ = 1.09, *p* = 0.352 for stroke patients; Accuracy: RM-ANOVA, *F*_(4, 44)_ = 0.82, *p* = 0.518 for able-bodied, and RM-ANOVA, *F*_(4, 20)_ = 0.39, *p* = 0.808 for stroke patients] (Figure 7).

## Discussion

Current rehabilitation therapies aim to restore motor function without integrating specific tasks for restoration of functional proprioception. In this study we demonstrated that 90 Hz, illusion-inducing vibrations can be used as an augmented proprioceptive sensory feedback to improve the motor performance of stroke patients. We provided several quantifiable measurements of the motor improvements (elbow extension, movement smoothness, movement directness, and Fitts' law parameters) and we identified a potential key role of proprioception in the motor recovery process. Finally, we compared the motor performance when agonist or antagonist muscles were stimulated, and we found that to promote better rehabilitative outcomes the vibration induced illusion of movements should be applied on the actively contracting, agonist muscle of repetitive movements.

Vibration has been used in therapy for stroke and has been shown to reduce movement time (Mortaza et al., 2019), but few studies have explored illusion-inducing vibration and its effect on task performance. Those that have (Conrad et al., 2011; Cordo et al., 2013; Rinderknecht et al., 2013) coupled vibration-induced illusions of movement with more standard sensorimotor rehabilitation therapies for post-stroke patients. Cordo et al. (2013) tested the motor recovery of stroke patients using robot-assisted cyclical hand movements with augmented proprioceptive feedback delivered to the antagonist muscles of the ongoing movement and with additional (torque or EMG) biofeedback. They found that at the end of the intervention period the patients improved the execution of voluntary upper limb movements (the motor impairment was assessed using the Stroke Impact Scale, the Upper Extremity Fugl-Meyer Assessment and the Box-and-Block Test). Because Cordo et al. used two feedback strategies concurrently and in addition to the robot-assisted therapy, it was not possible to separate the contribution of the three components to the motor recovery observed. Our study augments only proprioceptive feedback, separating its effects from other augmented feedback modalities. Contrary to the study by Cordo et al., the unaided task and the randomization of the different experimental conditions, ensured that our results were not caused by therapeutic effects, i.e., a continuous repetition of the same movement. Thus, our results suggest that the augmented proprioceptive feedback is the only parameter responsible for the motor improvements.

In another study (Conrad et al., 2011), stroke patients performed a pointing task with targets arranged around a circle while vibration was applied over the wrist flexors with the arm laying on a movable support and the hand grasping an handle equipped with force sensors. Vibration was delivered during the reaching phase independently from target position. Simultaneously EMG signals were recorded from the muscles of the whole arm. During the experiment the arm stability, muscle activity, and grip pressure at the target positions were measured. The results showed that the application of vibration produced a greater arm stability associated with a decreased muscle spasticity throughout the entire arm and a lower grip force. Analysis of the motor performance (i.e., kinematic evaluation of the reaching phase) was not carried out. The results we present here provide evidence that vibration-induced illusions of movement improve the ability of chronic stroke patients to perform voluntary movements.

In this study we demonstrated that vibration-induced illusions of movement applied over the triceps and biceps brachii significantly affected reaching directness and smoothness in stroke patients. In the absence of perturbations, a reaching task is characterized by a smooth and almost straight trajectory (Scott, 2004). A smooth movement has fewer motor commands due to the elimination of corrective and otherwise unnecessary sub-movements (i.e., a velocity with intermittent acceleration and deceleration) (Wolpert and Ghahramani, 2000; Balasubramanian et al., 2015). Stroke patients have difficulty performing smooth and coordinated movements (Rohrer et al., 2002; McCrea and Eng, 2005), and while it is unclear the neurophysiological process that leads the CNS to maximize the movement smoothness (Wolpert and Ghahramani, 2000; Balasubramanian et al., 2015), smoother movements are considered evidence of progressive motor recovery (Rohrer et al., 2002). In our experiment, we found that illusion-inducing vibration produced a smoother trajectory when applied over the triceps and not over the biceps brachii. This finding is supported by more direct trajectories and less divergence in the transverse plane (Figure 2B) when 90 Hz vibration was applied over the triceps compared to the biceps.

A reaching task or a rapid aiming task is composed of two phases: an initial impulse phase and then an online control phase in which sensory information is integrated to maximize the motor accuracy (Elliott et al., 2001; Goodman and Tremblay, 2018). During a reaching task these two phases are represented by an initial acceleration of the arm, followed by a deceleration to increase accuracy close to the target. The velocity peak between acceleration and deceleration divides the two phases (Wu et al., 2000). In our experiment, as in a previous study (Trombly, 1992) the velocity peak was reached between 30 and 50% of the trial duration and decreased with increased target distance. When targets at greater distances were presented, participants decelerated sooner. They likely did this to maintain movement accuracy for these more distant targets, possibly by increasing the amount of time they had to receive and interpret proprioceptive and tactile feedback. The timing of the velocity peak with respect to overall trial duration did not change between experimental conditions. This suggests that the augmented sensory feedback did not alter motor planning, i.e., the first phase of the reaching movement. As a consequence, kinematic improvements displayed by the participants depended more on the second phase of the movement. If participants were to complete a greater number of reaching repetitions, they might begin to slow their reach sooner as they learn, with the help of the sensory feedback, to better plan the movement.

In a previous study, reaching tests performed by able-bodied participants demonstrated that vibration applied over the antagonist muscle of the ongoing movement caused participants to undershoot the prescribed target, while no effects were observed when the same vibration was applied over the agonist muscle (Capaday and Cooke, 1983). Although we confirmed similar outcomes for antagonist muscles, we found larger elbow extension when vibration was applied over the agonist muscle. This result was obtained for both able-bodied and stroke patient participants. Capaday and Cooke (1983) suggested that target undershoots were caused by sensations of elongation in antagonist muscle stimulation as vibration applied over the lengthening antagonist muscle would misinform the participant about the real muscle extension, eliciting an apparent sensation that the arm was more extended than it truly was. With this proprioceptive misinformation, the participants wrongly believed that they had already reached the target. Although many studies endorse this perspective that vibrating the muscle induces an illusion of elongation of the vibrated muscle (Goodwin et al., 1972b; Roll and Vedel, 1982; Naito et al., 1999), our findings on the increased movement extension for both able-bodied and stroke participants when illusion-inducing vibration was applied to the triceps brachii indicate that vibration of the agonist muscle better matches predictions made by the internal model. Importantly, the stroke patient participants in this study demonstrated improvements even if they did not verbally report a clear illusion of movement. As reported in the introduction, targeted reinnervated amputees (Marasco et al., 2018) use illusion-inducing vibration of agonist muscles as feedback to improve performance. In amputees who have undergone targeted reinnervation, feedback from the skin is divorced from feedback from the underlying muscles. The change in reported direction of illusory movement for amputees with targeted reinnervation combined with improved performance with illusion-inducing vibration on the agonist muscle in this reaching task suggests that verbal reports of perceived movements do not necessarily match interpretations made by the internal model. This could indicate that a sensory group other than muscle spindles is responsible for providing movement feedback to the internal model, such as a rapidly adapting mechanoreceptor that projects to proprioceptive brain regions (Marasco et al., 2017). Alternatively, there may be a conflict between information provided by receptors in the skin and those in the muscles, which the internal model weights differently depending on the task.

In addition to the kinematic analysis, we quantified the motor performance using Fitts' law. McCrea and Eng (2005) previously used Fitts' law with stroke patients. However, unlike our study, they used the conventional linear regression version of Fitts' law (McCrea and Eng, 2005). In this study we used throughput instead because it combines the effects of intercept and slope of the regression version into a complete measure encompassing both the speed and accuracy of performance (Soukoreff and MacKenzie, 2004). Moreover, throughput is a more robust metric when a limited amount of data is available, as it was in our case (Soukoreff and MacKenzie, 2004; Thumser et al., 2018). Indeed, the six stroke patients enrolled the study and experimental protocol included only three repetitions of each target for each experimental condition. However, we were forced to limit the number of repetitions to three in order to avoid patient fatigue influencing motor performance and to reduce the experimental time.

The Fitts' law parameters reflected the divergence of the motor performance between able-bodied and stroke patients. In particular, the throughput indicated that, as expected, the able-bodied participants reached the more difficult targets in a shorter amount of time than the stroke patients. In addition, while there was no difference in the throughput across the experimental conditions for stroke patients, they performed better, although not statistically significantly so, when the triceps brachii was stimulated compared to other conditions. This result is supported by the other Fitts' parameters, which showed that stroke patients reached farther compared to no vibration and biceps vibration conditions, and in shorter amounts of time, compared to when the biceps were vibrated.

Unexpectedly, neither the Fitts' law parameters nor the kinematic results showed statistically significant differences between illusion-inducing and sham vibration, when the triceps brachii or when the biceps brachii were stimulated. Although we hypothesized this effect for the able-bodied participants, contrary to our expectations we found the same outcomes among stroke patients. Three different factors can be taken into consideration as a possible explanation: (i) presence of neuromotor noise, (ii) the actual state of the muscle (i.e., contracted or relaxed), and (iii) the presence of a less effective proprioceptive feedback delivered with 25 Hz vibration. McCrea and Eng (2005), combining the use of Fitts' law and kinematic measurements, suggested that stroke patients had greater neuromotor noise, which reduced the signal transmission capacity of motor commands and ultimately affected their motor performance. The neuromotor noise, combined with a limited number of repetitions to avoid excessive fatigue, could have limited the effects of inducing-illusion vibrations. Second, previous work reported that the level of contraction of a muscle influences the strength of the perceived illusion (Taylor et al., 2017). In particular, when a muscle is voluntarily contracted the perception of the illusion is decreased. In our case, voluntary muscle contractions, and the presence of muscle co-contractions and spasticity that usually impair stroke patients (Song and Tong, 2013), could have affected perception of the illusion-inducing vibration and reduced differences from sham vibration. Finally, results with sham vibration were often between those with no vibration and illusion-inducing vibration. It is possible that sham vibration provided a less vivid or less clearly perceived level of proprioceptive feedback that was available for use by the participants. Since few studies (Marasco et al., 2018) have compared the effects of sham and illusion-inducing vibration while participants were performing a voluntary controlled movement, more studies with a larger number of participants are needed to confirm or deny our results.

The outcomes of the reaching task and responses to augmented proprioceptive feedback via vibration-induced illusion of movement differed between able-bodied and stroke patient participants. Specifically, vibration conditions generally affected the stroke patients more strongly. Considering the task and the two groups of participants, it is reasonable to assume that the unimpaired and the impaired nervous systems integrated the augmented proprioceptive feedback with the other sensory modalities differently, which consequently changed motor control. To automatically accomplish even a simple movement, the central nervous system creates an internal model (or internal models) (Wolpert et al., 1998) that is used to estimate and adjust the position of our limbs via the integration of multi-modal sensory feedbacks, such as tactile, visual, and proprioceptive feedback. It has been suggested that uncertainty in sensory feedback affects the strength of the internal model (Shehata et al., 2018). Stroke patients are likely limited in their ability to make predictions with, and update, their internal model because brain lesions compromise communication between different areas, leading to artificial mismatches between motor intention, perceived actions, and actual motor execution. Moreover, compared to able-bodied individuals, the motor execution of stroke patients relies more on feedback control because of the greater neuromotor noise and the concomitant effects of several factors, such as abnormal muscle synergies, weakness, and spasticity, which require continuous adjustments in the motor trajectory (Ao et al., 2015). Integration of the vibratory feedback may have reinforced native sensory feedback information, thereby decreasing uncertainty in the feedback and strengthening the internal model, and consequently improving the motor performance. Interestingly, it was noted that stroke patients who present somatosensory deficits are more likely to be affected by extremity paresis, and that there is a negative correlation between the sensory impairment and both the rehabilitation time and the probability to reach significant motor improvements (Kessner et al., 2016). Emerging evidence from the last decades has shown that, although not all the patients respond similarly to the same sensory stimulation, specific sensory interventions (e.g., passive joint motion, electrical or thermal stimulations, pressure or cutaneous stimulation) improve sensory and motor capability, reduce spasticity, and reduce spatial neglect, and that these changes are associated with an increased cortical excitability and plastic modulations (Sullivan and Hedman, 2008).

In the same way, Seki and Fetz (2012) conducted animal experiments demonstrating that there is task-dependent afferent suppression at the spinal cord or at the cortical level during voluntary movement. In our experiment, two main sensory modalities were present, tactile and proprioceptive, while a vertical panel prevented the use of visual information. It is reasonable to believe that during motor execution able-bodied participants unconsciously gave priority to more robust sensory information (sensory feedback from cutaneous, tendon, joint, and other arm muscle receptors) and suppressed as noise the proprioceptive information provided by the augmented kinaesthetic feedback. In contrast, stroke patients were less capable of processing natural sensory feedback due to lesions in areas of the brain that process or transmit somatosensory information (Stewart et al., 2014) and due to increased neuromotor noise (McCrea and Eng, 2005). The motor improvements that we found suggest that the stroke-compromised central nervous system considered the augmented proprioceptive feedback to be an additional source of sensory feedback. This feedback was easily integrated with other sensory modalities to enhance movement perception by providing an additional source of feedback concurring with information provided by other sources, thus strengthening the internal model and improving performance.

Despite the kinematic improvements, some stroke patients reported strange and/or unclear movement sensations induced by the vibration at the beginning of the experiment. These differed from the expected elbow flexion or extension and varied from simple vibratory sensation perceived at a proximal location like the shoulder to perceived flexion or extension in more distal parts of the arm, such as the fingers. Similarly, in previous studies (Fusco et al., 2016; Beaulieu et al., 2020) stroke patients reported imprecise and unexpected illusory movement directions (Beaulieu et al., 2020) and patients with spinal cord injury reported the absence of movement illusion or the perception of strange sensations [e.g., some patients perceived that their arm was attempting to move against something or was hindered (Fusco et al., 2016)]. These unexpected sensations might be due to the reorganization of the sensorimotor networks at the spinal or cortical levels. In the anterior parietal cortex, the somatosensory areas have a specific topography of body parts, with the arm between the hand and the shoulder (Delhaye et al., 2018). Plastic changes after the injury might occur in both the affected as well as in remote brain areas, which may create new activity patterns involving areas other than the arm in the somatosensory topography (Di Pino et al., 2014). Such changes could disrupt the interpretation of the vibratory feedback and alter the resulting perceptions.

Future work is needed to corroborate our results in a larger number of participants and to evaluate whether active movement therapies integrating vibration-induced movement illusions on the agonist muscle of voluntarily performed movements as augmented feedback will promote long-lasting motor recovery. Previous studies demonstrated that the application of 90 Hz vibration activates not only the primary somatosensory area of the cortex (cytoarchitectonic areas 3a, 3b, and 1) but also the primary motor cortex, the dorsal premotor cortex, the supplementary motor area, and the cingulate motor area (Naito et al., 1999, 2007, 2016; Naito and Ehrsson, 2001). Activation of these areas can be used to increase the natural plasticity of the sensorimotor system (Di Pino et al., 2014) and the ability to “re-learn” patterns of activation with the final goal of improving motor recovery. The use of fMRI could confirm or deny the presence of significant long-lasting changes in the brain activity. Finally, benefits of vibration-induced illusions of movement will be tested with stroke patients in the chronic recovery phase in longer term trials as well as with stroke patients in the acute phase during rehabilitation.

## Data Availability Statement

The raw data supporting the conclusions of this article will be made available by the authors, without undue reservation. Requests to access the dataset should be directed to Paul D. Marasco, marascp2@ccf.org.

## Ethics Statement

The studies involving human participants were reviewed and approved by The Institutional Review Board of Cleveland Clinic. The patients/participants provided their written informed consent to participate in this study.

## Author Contributions

FF designed the software. FF, CS, and ZT collected the data. FF and CS analyzed the data. All the authors contributed to the design of the study first conceived by PM. All authors were involved in data interpretation, contributed to the manuscript, and approved the final version.

## Conflict of Interest

The authors declare that the research was conducted in the absence of any commercial or financial relationships that could be construed as a potential conflict of interest.

## Supplementary Material

Supplementary Video 1 Example of a reaching task trial for an able-bodied and a stroke patient participant.
