# Supplementary material for: Proprioceptive Augmentation With Illusory Kinaesthetic Sensation in Stroke Patients Improves Movement Quality in an Active Upper Limb Reach-and-Point Task
Source: Front Neurorobot. 2021 Mar 1;15:610673. doi: 10.3389/fnbot.2021.610673 (PMC7956990; doi:10.3389/fnbot.2021.610673)
Supplement: Supplementary file 2 [file Data_Sheet_1.pdf]

Supplementary Material

1 Supplementary Figures

The following three figures show the data distribution and the statistical differences when the linear mixed model reported a significant interaction effect between the target distance and the experimental condition for the able-bodied participants or the stroke patients. Supplementary Figure 1 and Supplementary Figure 2 refer to kinematic measurements while Supplementary Figure 3 refers to Fitts' Law parameters.

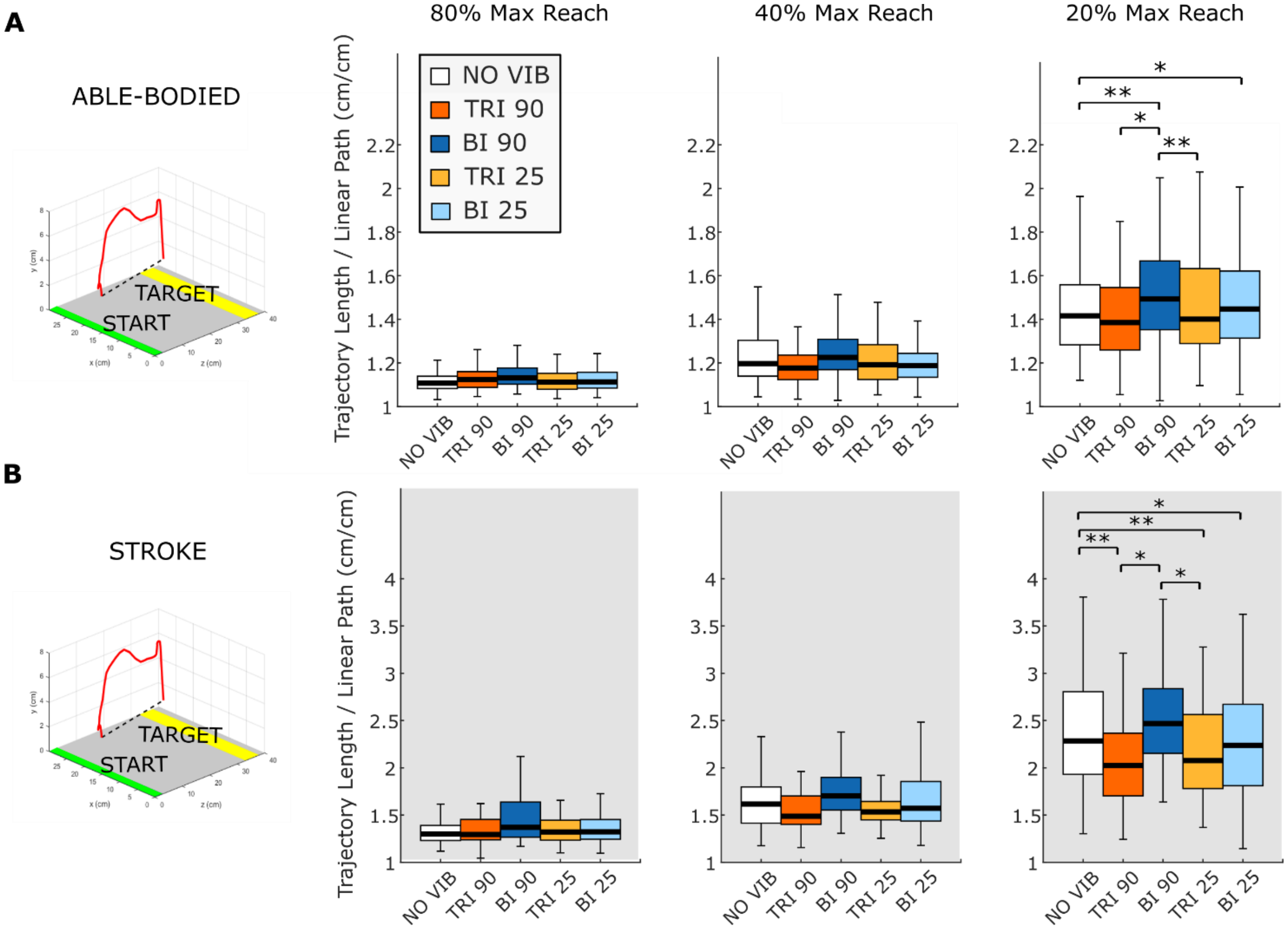

Supplementary Figure 1: Directness of the trajectory for both (A) able-bodied and (B) stroke patients showed a significant interaction effect (vibration condition*target distance). The directness was calculated as the ratio between the length of the travelled trajectory and the length of a linear path connecting the initial and the final position. In each subplot data are reported for targets at 80% (left), 40% (middle) and 20% (right) of the maximum reachable distance. Medians and interquartile ranges [25th and 75th percentile (Q25, Q75)] with whiskers indicating the range of non-outlier values are shown for data aggregated across all participants for both able-bodied and stroke patients when no

vibration (white, NO VIB), 90 Hz vibration the triceps (orange, TRI 90), 90 Hz vibration on the biceps (blue, BI 90), 25 Hz vibration on the triceps (yellow, TRI 25), and 25 Hz vibration on the biceps (light blue, BI 25) was applied. Significant differences between vibration conditions were present only for targets at 20% of the maximum reachable distance indicating that there were less direct trajectories with no vibration and with vibrations on the biceps brachii. Similar but non-significant differences were found for the targets presented at 40% and 80% of the maximum reachable distance. *p<0.05, ** p<0.001.

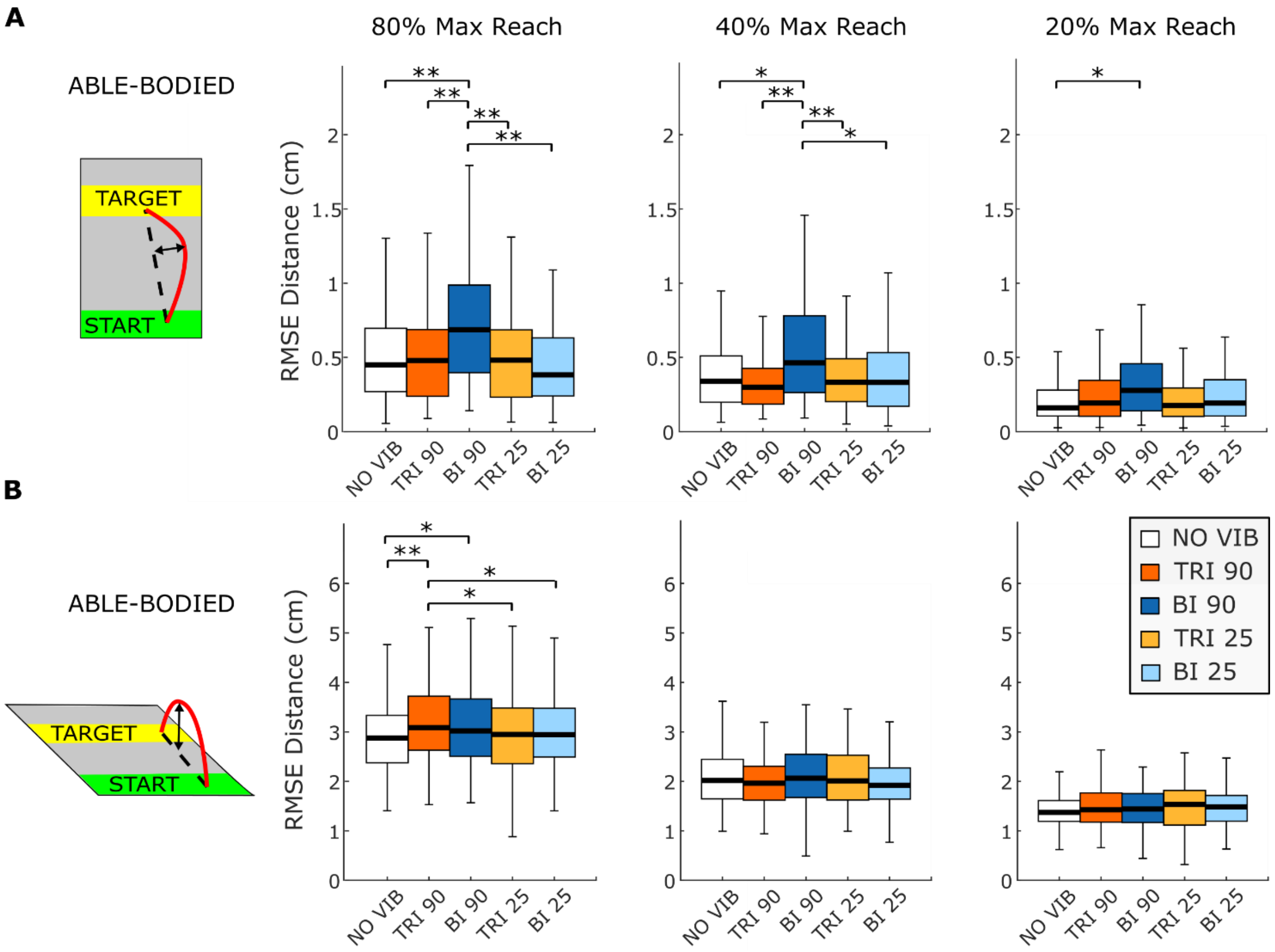

Supplementary Figure 2: The root mean square errors (RMSE) of the distance of the trajectory from a linear path connecting the initial and the final position showed a significant interaction effect in (A) the transverse plane and in (B) the sagittal plane. In each subplot data are reported for targets at 80% (left), 40% (middle) and 20% (right) of the maximum reachable distance. Medians and interquartile ranges [25th and 75th percentile (Q25, Q75)] with whiskers indicating the range of non-outlier values are shown for data aggregated across all participants when no vibration (white, NO VIB), 90 Hz vibration the triceps (orange, TRI 90), 90 Hz vibration on the biceps (blue, BI 90), 25 Hz vibration on the triceps (yellow, TRI 25), and 25 Hz vibration on the biceps (light blue, BI 25) was applied. In the horizontal plane differences between experimental conditions showed that as the distance increased, the 90Hz vibration on the biceps produced a higher divergence. On the other hand, in the vertical plane

the higher divergence was produced by both 90Hz vibrations on triceps and biceps only at 80% of the maximum reachable distance. *p<0.05, ** p<0.001.

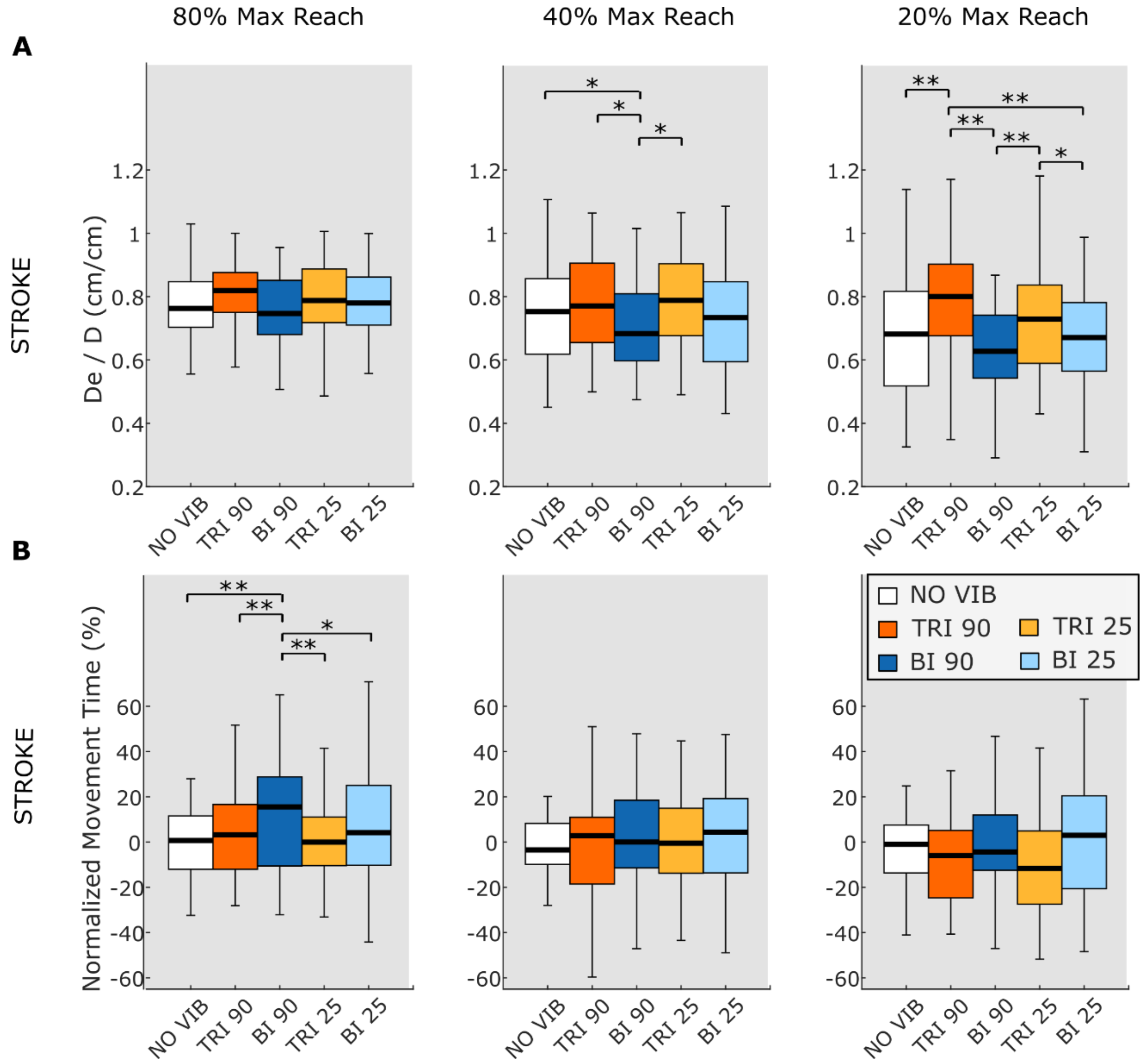

Supplementary Figure 3: Two Fitts' Law parameters, (A) the ratio between the effective and the prescribed distance (De/D) and (B) the normalized movement time, showed significant interaction effects (vibration condition*target distance) only in stroke patients. In each subplot data are reported for targets at 80% (left), 40% (middle) and 20% (right) of the maximum reachable distance. Medians and interquartile ranges [25th and 75th percentile (Q25, Q75)] with whiskers indicating the range of non-outlier values are shown for data aggregated across all stroke patients when no vibration (white, NO VIB), 90 Hz vibration the triceps (orange, TRI 90), 90 Hz vibration on the biceps (blue, BI 90), 25 Hz vibration on the triceps (yellow, TRI 25), and 25 Hz vibration on the biceps (light blue, BI 25) was applied. The distances ratio (De/D) reported significant differences between experimental conditions only at the 20% and 40% of the maximum reachable distance indicating that vibration on the triceps brachii allowed stroke patients to better approach the target. Although non-significant a similar trend was also observed at the 80% of the maximum reachable distance. On the other hand, the normalized

movement time showed that the movement time significantly increased when the biceps was vibrated at 90Hz only at the 80% of the maximum reachable distance. *p<0.05, ** p<0.001.

2 Results of the Linear Mixed Model Analyses

In the following tables, *p*-values (Sig.) for main and interaction effects are presented for kinematics and Fitts' Law measures. Mean differences (Mean Diff.), standard errors (Std. Error) and *p*-values for Bonferroni-corrected post-hoc pairwise comparisons are also shown. ** indicates that a Bonferroni adjustment for multiple comparisons was made.

Example SPSS syntax for these analyses, where *Measure* is replaced by the name of the measure to be analyzed and post-hoc pairwise comparisons (/EMMEANS) are only examined if the related main or interaction effect was significant:

```
MIXED ID BY Vibration_Condition Target_Distance Target_Width
/CRITERIA=CIN(95) MXITER(100) MXSTEP(10) SCORING(1)
SINGULAR(0.000000000001) HCONVERGE(0, ABSOLUTE) LCONVERGE(0, ABSOLUTE)
PCONVERGE(0.000001, ABSOLUTE)
/FIXED= Vibration_Condition Target_Distance Vibration_Condition *
Target_Distance  | SSTYPE(3)
/METHOD=REML
/RANDOM=INTERCEPT | SUBJECT(SubjNum) COVTYPE(ID)
/REPEATED=TrialNum | SUBJECT(SubjNum* Vibration_Condition *
Target_Distance *Target_Width) COVTYPE(DIAG)
/EMMEANS=TABLES(Vibration_Condition) COMPARE ADJ(BONFERRONI)
/EMMEANS=TABLES(Target_Distance) COMPARE ADJ(BONFERRONI)
/EMMEANS=TABLES(Vibration_Condition * Target_Distance)
COMPARE(Vibration_Condition) ADJ(BONFERRONI)
/EMMEANS=TABLES(Vibration_Condition * Target_Distance)
COMPARE(Target_Distance) ADJ(BONFERRONI).
```

2.1 Kinematics

2.1.1 Directness

2.1.1.1 Able-Bodied

2.1.1.1.1 Tests of Fixed Main and Interaction Effects

Effect	Numerator df	Denominator df	F	Sig.
Intercept	1	10.993	3489.154	0.000
Vibration condition	4	1099.569	5.807	0.000
Target Distance	2	1099.571	417.455	0.000
Experimental Condition * Target Distance	8	1099.570	2.220	0.024

2.1.1.1.2 Pairwise Comparisons

(I)		(J)	Mean Diff. (I-J)	Std. Error	df	Sig.**
Vibration Condition						
BI 90		BI 25	0.042	0.018	1098.768	0.171
		NO VIB	0.073	0.018	1100.002	0.000
		TRI 25	0.064	0.018	1100.002	0.003
		TRI 90	0.070	0.018	1099.695	0.001
BI 25		NO VIB	0.031	0.018	1099.074	0.849
		TRI 25	0.021	0.018	1099.074	1.000
		TRI 90	0.027	0.018	1098.768	1.000
NO VIB		TRI 25	-0.009	0.018	1100.309	1.000
		TRI 90	-0.004	0.018	1100.002	1.000
TRI 25		TRI 90	0.006	0.018	1100.002	1.000
Target Distance						
20%		40%	0.292	0.014	1099.201	0.000
		80%	0.380	0.014	1099.386	0.000
40%		80%	0.088	0.014	1100.125	0.000
Vibration Condition * Target Distance						
20%	BI 90	BI 25	0.059	0.031	1095.677	0.559
		NO VIB	0.159	0.031	1099.389	0.000
		TRI 25	0.129	0.031	1099.389	0.000
		TRI 90	0.122	0.031	1099.389	0.001
20%	BI 25	NO VIB	0.100	0.031	1096.588	0.013

		TRI 25	0.069	0.031	1096.588	0.247
		TRI 90	0.063	0.031	1096.588	0.425
20%	NO VIB	TRI 25	-0.030	0.031	1100.309	1.000
		TRI 90	-0.037	0.031	1100.309	1.000
20%	TRI 25	TRI 90	-0.007	0.031	1100.309	1.000
40%	BI 90	BI 25	0.052	0.031	1100.309	0.910
		NO VIB	0.028	0.031	1100.309	1.000
		TRI 25	0.046	0.031	1100.309	1.000
		TRI 90	0.068	0.031	1099.389	0.279
40%	BI 25	NO VIB	-0.024	0.031	1100.309	1.000
		TRI 25	-0.006	0.031	1100.309	1.000
		TRI 90	0.016	0.031	1099.389	1.000
40%	NO VIB	TRI 25	0.018	0.031	1100.309	1.000
		TRI 90	0.039	0.031	1099.389	1.000
40%	TRI 25	TRI 90	0.022	0.031	1099.389	1.000
80%	BI 90	BI 25	0.016	0.031	1100.309	1.000
		NO VIB	0.032	0.031	1100.309	1.000
		TRI 25	0.017	0.031	1100.309	1.000
		TRI 90	0.019	0.031	1100.309	1.000
80%	BI 25	NO VIB	0.016	0.031	1100.309	1.000
		TRI 25	0.001	0.031	1100.309	1.000
		TRI 90	0.003	0.031	1100.309	1.000
80%	NO VIB	TRI 25	-0.016	0.031	1100.309	1.000
		TRI 90	-0.013	0.031	1100.309	1.000
80%	TRI 25	TRI 90	0.002	0.031	1100.309	1.000

2.1.1.2 Stroke Patients

2.1.1.2.1 Tests of Fixed Main and Interaction Effects

Effect	Numerator df	Denominator df	F	Sig.
Intercept	1	4.982	357.882	0.000
Vibration condition	4	731.821	5.417	0.000
Target Distance	2	731.835	232.318	0.000
Experimental Condition * Target Distance	8	731.660	2.264	0.021

2.1.1.2.2 Pairwise Comparisons

(I)		(J)	Mean Diff. (I-J)	Std. Error.	df	Sig.**
Vibration Condition						
BI 90		BI 25	0.062	0.063	735.285	1.000
		NO VIB	0.190	0.063	734.981	0.026
		TRI 25	0.190	0.063	734.981	0.026
		TRI 90	0.176	0.063	735.543	0.052
BI 25		NO VIB	-0.102	0.063	728.745	1.000
		TRI 25	0.129	0.063	730.423	0.412
		TRI 90	0.115	0.063	731.090	0.680
NO VIB		TRI 25	0.231	0.063	728.716	0.003
		TRI 90	0.217	0.063	729.370	0.006
TRI 25		TRI 90	-0.014	0.063	730.955	1.000
Target Distance						
20%		40%	0.759	0.049	734.038	0.000
		80%	1.005	0.049	730.031	0.000
40%		80%	0.247	0.049	731.589	0.000
Vibration Condition * Target Distance						
20%	BI 90	BI 25	0.108	0.108	734.312	1.000
		NO VIB	-0.225	0.109	732.704	0.389
		TRI 25	0.316	0.108	734.312	0.037
		TRI 90	0.308	0.109	738.684	0.048
20%	BI 25	NO VIB	-0.333	0.108	727.620	0.022
		TRI 25	0.209	0.108	729.390	0.532
		TRI 90	0.201	0.108	734.312	0.643
20%	NO VIB	TRI 25	0.541	0.108	727.620	0.000
		TRI 90	0.534	0.109	732.704	0.000
20%	TRI 25	TRI 90	-0.008	0.108	734.312	1.000
40%	BI 90	BI 25	0.030	0.109	739.480	1.000
		NO VIB	-0.002	0.109	739.480	1.000
		TRI 25	0.148	0.110	743.281	1.000
		TRI 90	0.124	0.109	739.480	1.000
40%	BI 25	NO VIB	-0.032	0.108	729.390	1.000

		TRI 25	0.118	0.108	734.312	1.000
		TRI 90	0.094	0.108	729.390	1.000
40%	NO VIB	TRI 25	0.150	0.108	734.312	1.000
		TRI 90	0.125	0.108	729.390	1.000
40%	TRI 25	TRI 90	-0.025	0.108	734.312	1.000
80%	BI 90	BI 25	0.047	0.109	730.933	1.000
		NO VIB	0.105	0.108	727.620	1.000
		TRI 25	0.106	0.109	725.453	1.000
		TRI 90	0.096	0.108	727.620	1.000
80%	BI 25	NO VIB	0.058	0.110	728.890	1.000
		TRI 25	0.059	0.110	726.466	1.000
		TRI 90	0.050	0.110	728.890	1.000
80%	NO VIB	TRI 25	0.001	0.109	723.368	1.000
		TRI 90	-0.009	0.109	725.689	1.000
80%	TRI 25	TRI 90	-0.009	0.109	723.368	1.000

2.1.2 Root Mean Square Errors of the Trajectory Deviation in the Transverse Plane

2.1.2.1 Able-Bodied

2.1.2.1.1 Tests of Fixed Main and Interaction Effects

Effect	Numerator df	Denominator df	F	Sig.
Intercept	1	10.980	94.534	0.000
Vibration condition	4	1548.678	23.430	0.000
Target Distance	2	1548.684	135.097	0.000
Experimental Condition * Target Distance	8	1548.676	2.485	0.011

2.1.2.1.2 Pairwise Comparisons

(I)	(J)	Mean Diff. (I-J)	Std. Error.	df	Sig.**
Vibration Condition					
BI 90	BI 25	0.177	0.023	1548.217	0.000
	NO VIB	0.169	0.023	1548.723	0.000
	TRI 25	0.172	0.023	1548.723	0.000
	TRI 90	0.175	0.023	1547.920	0.000

BI 25		NO VIB	-0.008	0.023	1549.025	1.000
		TRI 25	-0.005	0.023	1549.025	1.000
		TRI 90	-0.002	0.023	1548.217	1.000
NO VIB		TRI 25	0.002	0.023	1549.514	1.000
		TRI 90	0.006	0.023	1548.723	1.000
TRI 25		TRI 90	0.004	0.023	1548.723	1.000
Target Distance						
20%		40%	-0.149	0.018	1548.263	0.000
		80%	-0.289	0.018	1548.748	0.000
40%		80%	-0.140	0.018	1549.041	0.000
Vibration Condition * Target Distance						
20%	BI 90	BI 25	0.087	0.040	1545.407	0.279
		NO VIB	0.126	0.039	1547.110	0.014
		TRI 25	0.094	0.039	1547.110	0.172
		TRI 90	0.075	0.039	1547.110	0.551
20%	BI 25	NO VIB	0.039	0.039	1547.935	1.000
		TRI 25	0.007	0.039	1547.935	1.000
		TRI 90	-0.012	0.039	1547.935	1.000
20%	NO VIB	TRI 25	-0.032	0.039	1549.514	1.000
		TRI 90	-0.050	0.039	1549.514	1.000
20%	TRI 25	TRI 90	-0.018	0.039	1549.514	1.000
40%	BI 90	BI 25	0.159	0.039	1549.514	0.001
		NO VIB	0.132	0.039	1549.514	0.008
		TRI 25	0.163	0.039	1549.514	0.000
		TRI 90	0.197	0.039	1547.110	0.000
40%	BI 25	NO VIB	-0.027	0.039	1549.514	1.000
		TRI 25	0.004	0.039	1549.514	1.000
		TRI 90	0.037	0.039	1547.110	1.000
40%	NO VIB	TRI 25	0.031	0.039	1549.514	1.000
		TRI 90	0.064	0.039	1547.110	1.000
40%	TRI 25	TRI 90	0.034	0.039	1547.110	1.000
80%	BI 90	BI 25	0.285	0.039	1549.514	0.000
		NO VIB	0.250	0.039	1549.514	0.000
		TRI 25	0.259	0.039	1549.514	0.000

		TRI 90	0.254	0.039	1549.514	0.000
80%	BI 25	NO VIB	-0.035	0.039	1549.514	1.000
		TRI 25	-0.027	0.039	1549.514	1.000
		TRI 90	-0.031	0.039	1549.514	1.000
80%	NO VIB	TRI 25	0.008	0.039	1549.514	1.000
		TRI 90	0.004	0.039	1549.514	1.000
80%	TRI 25	TRI 90	-0.004	0.039	1549.514	1.000

2.1.2.2 Stroke Patients

2.1.2.2.1 Tests of Fixed Main and Interaction Effects

Effect	Numerator df	Denominator df	F	Sig.
Intercept	1	4.994	42.412	0.001
Vibration condition	4	758.968	4.243	0.002
Target Distance	2	758.943	113.091	0.000
Experimental Condition * Target Distance	8	758.764	0.629	0.754

2.1.2.2.2 Pairwise Comparisons

(I)	(J)	Mean Diff. (I-J)	Std. Error.	df	Sig.**
Vibration Condition					
BI 90	BI 25	0.070	0.062	756.775	1.000
	NO VIB	0.129	0.062	757.569	0.383
	TRI 25	0.177	0.062	756.893	0.046
	TRI 90	0.233	0.062	755.656	0.002
BI 25	NO VIB	0.059	0.062	761.284	1.000
	TRI 25	0.107	0.062	760.607	0.870
	TRI 90	0.163	0.062	759.529	0.093
NO VIB	TRI 25	0.048	0.062	761.254	1.000
	TRI 90	0.103	0.062	760.225	0.970
TRI 25	TRI 90	0.056	0.062	759.558	1.000
Target Distance					
20%	40%	-0.222	0.048	756.099	0.000
	80%	-0.712	0.048	760.983	0.000
40%	80%	-0.489	0.048	759.981	0.000

2.1.3 Root Mean Square Errors of the Trajectory Deviation in The Sagittal Plane

2.1.3.1 Able-Bodied

2.1.3.1.1 Tests of Fixed Main and Interaction Effects

Effect	Numerator df	Denominator df	F	Sig.
Intercept	1	10.999	281.488	0.000
Vibration condition	4	1582.661	3.451	0.008
Target Distance	2	1582.667	1396.412	0.000
Experimental Condition * Target Distance	8	1582.658	2.905	0.003

2.1.3.1.2 Pairwise Comparisons

(I)		(J)	Mean Diff. (I-J)	Std. Error.	df	Sig.**
Vibration Condition						
BI 90		BI 25	0.101	0.038	1582.139	0.084
		NO VIB	0.116	0.038	1582.870	0.024
		TRI 25	0.053	0.038	1582.870	1.000
		TRI 90	0.020	0.038	1582.439	1.000
BI 25		NO VIB	0.015	0.038	1582.578	1.000
		TRI 25	-0.047	0.038	1582.578	1.000
		TRI 90	-0.081	0.038	1582.139	0.340
NO VIB		TRI 25	-0.063	0.038	1583.287	1.000
		TRI 90	-0.096	0.038	1582.870	0.117
TRI 25		TRI 90	-0.033	0.038	1582.870	1.000
Target Distance						
20%		40%	-0.561	0.030	1582.350	0.000
		80%	-1.540	0.030	1582.613	0.000
40%		80%	-0.979	0.029	1583.038	0.000
Vibration Condition * Target Distance						
20%	BI 90	BI 25	0.047	0.066	1579.556	1.000
		NO VIB	0.103	0.066	1581.996	1.000
		TRI 25	-0.003	0.066	1581.996	1.000
		TRI 90	0.055	0.066	1581.996	1.000
20%	BI 25	NO VIB	0.056	0.066	1581.027	1.000
		TRI 25	-0.050	0.066	1581.027	1.000

		TRI 90	0.008	0.066	1581.027	1.000
20%	NO VIB	TRI 25	-0.106	0.066	1583.287	1.000
		TRI 90	-0.048	0.066	1583.287	1.000
20%	TRI 25	TRI 90	0.058	0.066	1583.287	1.000
40%	BI 90	BI 25	0.162	0.066	1583.287	0.138
		NO VIB	0.046	0.066	1583.287	1.000
		TRI 25	0.060	0.066	1583.287	1.000
		TRI 90	0.120	0.066	1581.996	0.691
40%	BI 25	NO VIB	-0.117	0.066	1583.287	0.764
		TRI 25	-0.102	0.066	1583.287	1.000
		TRI 90	-0.042	0.066	1581.996	1.000
40%	NO VIB	TRI 25	0.015	0.066	1583.287	1.000
		TRI 90	0.075	0.066	1581.996	1.000
40%	TRI 25	TRI 90	0.060	0.066	1581.996	1.000
80%	BI 90	BI 25	0.093	0.066	1583.287	1.000
		NO VIB	0.199	0.066	1583.287	0.025
		TRI 25	0.103	0.066	1583.287	1.000
		TRI 90	-0.115	0.066	1583.287	0.801
80%	BI 25	NO VIB	0.106	0.066	1583.287	1.000
		TRI 25	0.010	0.066	1583.287	1.000
		TRI 90	-0.208	0.066	1583.287	0.016
80%	NO VIB	TRI 25	-0.096	0.066	1583.287	1.000
		TRI 90	-0.315	0.066	1583.287	0.000
80%	TRI 25	TRI 90	-0.218	0.066	1583.287	0.009

2.1.3.2 Stroke Patients

2.1.3.2.1 Tests of Fixed Main and Interaction Effects

Effect	Numerator df	Denominator df	F	Sig.
Intercept	1	4.997	104.735	0.000
Vibration condition	4	717.139	1.581	0.177
Target Distance	2	717.085	200.223	0.000
Experimental Condition * Target Distance	8	717.046	0.612	0.768

2.1.3.2.2 Pairwise Comparisons

(I)	(J)	Mean Diff. (I-J)	Std. Error.	df	Sig.**
Target Distance					
20%	40%	-0.400	0.069	720.792	0.000
	80%	-1.342	0.069	714.442	0.000
40%	80%	-0.942	0.069	716.125	0.000

2.1.4 Smoothness

2.1.4.1 Able-Bodied

2.1.4.1.1 Tests of Fixed Main and Interaction Effects

Effect	Numerator df	Denominator df	F	Sig.
Intercept	1	11.000	628.721	0.000
Vibration condition	4	1580.428	1.317	0.262
Target Distance	2	1580.461	1081.245	0.000
Experimental Condition * Target Distance	8	1580.405	0.703	0.689

2.1.4.1.2 Pairwise Comparisons

(I)	(J)	Mean Diff. (I-J)	Std. Error.	df	Sig.**
Target Distance					
20%	40%	-0.076	0.003	1580.494	0.000
	80%	-0.117	0.003	1580.533	0.000
40%	80%	-0.041	0.003	1580.363	0.000

2.1.4.2 Stroke Patients

2.1.4.2.1 Tests of Fixed Main and Interaction Effects

Effect	Numerator df	Denominator df	F	Sig.
Intercept	1	5.000	426.689	0.000
Vibration condition	4	773.725	3.049	0.016
Target Distance	2	773.658	335.994	0.000
Experimental Condition * Target Distance	8	773.567	1.018	0.421

2.1.4.2.2 Pairwise Comparisons

(I)	(J)	Mean Diff. (I-J)	Std. Error.	df	Sig.**
Vibration Condition					
BI 90	BI 25	-0.009	0.005	774.603	0.655
	NO VIB	-0.011	0.005	774.387	0.221
	TRI 25	-0.011	0.005	774.265	0.287
	TRI 90	-0.017	0.005	774.872	0.007
BI 25	NO VIB	-0.002	0.005	772.982	1.000
	TRI 25	-0.002	0.005	772.898	1.000
	TRI 90	-0.008	0.005	773.701	1.000
NO VIB	TRI 25	0.000	0.005	772.583	1.000
	TRI 90	-0.005	0.005	773.403	1.000
TRI 25	TRI 90	-0.006	0.005	773.324	1.000
Target Distance					
20%	40%	-0.057	0.004	774.574	0.000
	80%	-0.097	0.004	773.169	0.000
40%	80%	-0.040	0.004	773.369	0.000

2.1.5 Movement Extension (Elbow Angle)

2.1.5.1 Able-Bodied

2.1.5.1.1 Tests of Fixed Main and Interaction Effects

Effect	Numerator df	Denominator df	F	Sig.
Intercept	1	10.978	582.362	0.000
Vibration condition	4	1583.352	3.056	0.016
Target Distance	2	1583.357	14399.439	0.000
Experimental Condition * Target Distance	8	1583.342	0.856	0.553

2.1.5.1.2 Pairwise Comparisons

(I)	(J)	Mean Diff. (I-J)	Std. Error.	df	Sig.**
Vibration Condition					
BI 90	BI 25	-0.563	0.384	1583.692	1,000
	NO VIB	-0.780	0.383	1583.261	0.418
	TRI 25	-0.827	0.380	1583.261	0.308
	TRI 90	-1.305	0.383	1583.650	0.007
BI 25	NO VIB	-0.216	0.383	1583.314	1.000
	TRI 25	-0.264	0.383	1583.314	1.000
	TRI 90	-0.741	0.384	1583.692	0.535
NO VIB	TRI 25	-0.047	0.382	1582.856	1.000
	TRI 90	-0.525	0.383	1583.261	1.000
TRI 25	TRI 90	-0.478	0.383	1583.261	1.000
Target Distance					
20%	40%	-14.940	0.297	1583.602	0.000
	80%	-49.095	0.297	1583.372	0.000
40%	80%	-34.156	0.296	1583.101	0.000

2.1.5.2 Stroke Patients

2.1.5.2.1 Tests of Fixed Main and Interaction Effects

Effect	Numerator df	Denominator df	F	Sig.
Intercept	1	3.999	42.755	0.003
Vibration condition	4	637.695	10.090	0.000
Target Distance	2	637.621	1432.624	0.000
Experimental Condition * Target Distance	8	637.428	1.711	0.093

2.1.5.2.2 Pairwise Comparisons

(I)	(J)	Mean Diff. (I-J)	Std. Error.	df	Sig.**
Vibration Condition					
BI 90	BI 25	-2.217	0.779	637.907	0.046
	NO VIB	-3.034	0.778	637.616	0.001
	TRI 25	-3.864	0.779	637.830	0.000
	TRI 90	4.498	0.777	637.362	0.000
BI 25	NO VIB	-0.817	0.778	637.622	1.000

	TRI 25	-1.647	0.779	637.985	0.349
	TRI 90	2.281	0.778	637.707	0.035
NO VIB	TRI 25	-0.830	0.777	637.564	1.000
	TRI 90	-1.464	0.776	637.305	0.596
TRI 25	TRI 90	-0.634	0.777	637.635	1.000
Target Distance					
20%	40%	-11.788	0.600	637.005	0.000
	80%	-31.971	0.604	637.997	0.000
40%	80%	-20.182	0.604	638.104	0.000

2.1.6 Movement Extension (Normalized Elbow Angle)

2.1.6.1 Able-Bodied

2.1.6.1.1 Tests of Fixed Main and Interaction Effects

Effect	Numerator df	Denominator df	F	Sig.
Intercept	1	10.978	0.176	0.683
Vibration condition	4	1573.772	4.590	0.001
Target Distance	2	1573.777	5.297	0.005
Experimental Condition * Target Distance	8	1573.758	1.310	0.234

2.1.6.1.2 Pairwise Comparisons

(I)	(J)	Mean Diff. (I-J)	Std. Error.	df	Sig.**
Vibration Condition					
BI 90	BI 25	-0.829	1.324	1574.651	1.000
	NO VIB	-1.411	1.320	1573.443	1.000
	TRI 25	-2.005	1.320	1573.443	1.000
	TRI 90	-5.221	1.321	1574.186	0.001
BI 25	NO VIB	-0.581	1.322	1573.918	1.000
	TRI 25	-1.176	1.322	1573.918	1.000
	TRI 90	-4.392	1.324	1574.651	0.009
NO VIB	TRI 25	-0.594	1.319	1572.683	1.000
	TRI 90	-3.810	1.320	1573.443	0.040
TRI 25	TRI 90	-3.216	1.320	1573.443	0.150
Target Distance					
20%	40%	-1.920	1.024	1574.319	0.183
	80%	1.393	1.024	1573.875	0.521
40%	80%	3.313	1.022	1573.141	0.004

2.1.6.2 Stroke Patients

2.1.6.2.1 Tests of Fixed Main and Interaction Effects

Effect	Numerator df	Denominator df	F	Sig.
Intercept	1	3.967	0.150	0.719
Vibration condition	4	569.535	5.794	0.000
Target Distance	2	569.513	0.021	0.980
Experimental Condition * Target Distance	8	569.455	0.275	0.974

2.1.6.2.2 Pairwise Comparisons

(I)	(J)	Mean Diff. (I-J)	Std. Error.	df	Sig.**
Vibration Condition					
BI 90	BI 25	-9.337	4.435	572.381	0.357
	NO VIB	-8.448	4.423	571.497	0.566
	TRI 25	-16.874	4.433	572.349	0.002
	TRI 90	-18.940	4.429	573.654	0.000
BI 25	NO VIB	0.889	4.408	566.427	1.000

	TRI 25	-7.537	4.420	567.513	0.887
	TRI 90	-9.603	4.415	568.807	0.300
NO VIB	TRI 25	-8.426	4.408	566.538	0.564
	TRI 90	-10.492	4.404	567.834	0.175
TRI 25	TRI 90	-2.066	4.415	568.867	1.000

2.1.7 Velocity Peak

2.1.7.1 Able-Bodied

2.1.7.1.1 Tests of Fixed Main and Interaction Effects

**Effect**	**Numerator df**	**Denominator df**	**F**	**Sig.**
Intercept	1	10.984	413.795	0.000
Vibration condition	4	1588.304	0.294	0.882
Target Distance	2	1588.310	3739.563	0.000
Experimental Condition * Target Distance	8	1588.298	0.564	0.808

2.1.7.1.2 Pairwise Comparisons

(I)	(J)	Mean Diff. (I-J)	Std. Error.	df	Sig.**
Target Distance					
20%	40%	-23.223	0.732	1588.203	0.000
	80%	-62.582	0.732	1588.297	0.000
40%	80%	-39.358	0.731	1588.432	0.000

2.1.7.2 Stroke Patients

2.1.7.2.1 Tests of Fixed Main and Interaction Effects

**Effect**	**Numerator df**	**Denominator df**	**F**	**Sig.**
Intercept	1	5.002	404.970	0.000
Vibration condition	4	743.562	1.836	0.120
Target Distance	2	743.499	191.934	0.000
Experimental Condition * Target Distance	8	743.482	1.095	0.364

2.1.7.2.2 Pairwise Comparisons

(I)	(J)	Mean Diff. (I-J)	Std. Error.	df	Sig.**
Target Distance					
20%	40%	-5.762	0.870	746.520	0.000
	80%	-16.809	0.872	741.464	0.000
40%	80%	-11.046	0.872	742.620	0.000

2.1.8 Time to Velocity Peak

2.1.8.1 Able-Bodied

2.1.8.1.1 Tests of Fixed Main and Interaction Effects

**Effect**	**Numerator df**	**Denominator df**	**F**	**Sig.**
Intercept	1	11.000	285,567	0.000
Vibration condition	4	1563.929	0. 526	0.717
Target Distance	2	1563.933	18.625	0.000
Experimental Condition * Target Distance	8	1563.915	0.658	0.729

2.1.8.1.2 Pairwise Comparisons

(I)	(J)	Mean Diff. (I-J)	Std. Error.	df	Sig.**
Target Distance					
20%	40%	-0.012	0.008	1564.532	0.394
	80%	-0.47	0.008	1564.101	0.000
40%	80%	-0.35	0.008	1563.174	0.000

2.1.8.2 Stroke Patients

2.1.8.2.1 Tests of Fixed Main and Interaction Effects

**Effect**	**Numerator df**	**Denominator df**	**F**	**Sig.**
Intercept	1	5.001	38.112	0.002
Vibration condition	4	761.922	0.636	0.637
Target Distance	2	761.830	10.129	0.000
Experimental Condition * Target Distance	8	761.770	1.750	0.084

2.1.8.2.2 Pairwise Comparisons

(I)	(J)	Mean Diff. (I-J)	Std. Error.	df	Sig.**
Target Distance					
20%	40%	-0.012	0.029	760.814	1.000
	80%	-1.07	0.029	762.751	0.001
40%	80%	-0.119	0.029	762.104	0.000

2.1.9 Normalized Time to Velocity Peak

2.1.9.1 Able-Bodied

2.1.9.1.1 Tests of Fixed Main and Interaction Effects

Effect	Numerator df	Denominator df	F	Sig.
Intercept	1	10.980	26.119	0.000
Vibration condition	4	1581.487	2.184	0.069
Target Distance	2	1581.487	3.041	0.048
Experimental Condition * Target Distance	8	1581.478	0.940	0.482

2.1.9.1.2 Pairwise Comparisons

(I)	(J)	Mean Diff. (I-J)	Std. Error.	df	Sig.**
Target Distance					
20%	40%	9.669	4.612	1581.474	0.109
	80%	10.028	4.610	1581.558	0.089
40%	80%	0.360	4.603	1581.431	1.000

2.1.9.2 Stroke Patients

2.1.9.2.1 Tests of Fixed Main and Interaction Effects

Effect	Numerator df	Denominator df	F	Sig.
Intercept	1	4.967	3.154	0.136
Vibration condition	4	724.259	1.736	0.140
Target Distance	2	724.189	4.944	0.007
Experimental Condition * Target Distance	8	724.216	0.801	0.602

2.1.9.2.2 Pairwise Comparisons

(I)	(J)	Mean Diff. (I-J)	Std. Error.	df	Sig.**
Target Distance					
20%	40%	-8.148	8.539	726.344	1.000
	80%	-26.308	8.561	723.059	0.007
40%	80%	-18.160	8.563	723.253	0.103

2.2 Fitts' Law Parameters

2.2.1 The Ratio Between the Expected Over the Prescribed Target Distance (De/D)

2.2.1.1 Able-Bodied

2.2.1.1.1 Tests of Fixed Main and Interaction Effects

Effect	Numerator df	Denominator df	F	Sig.
Intercept	1	10.984	1082.527	0.000
Vibration condition	4	1541.726	5.429	0.000
Target Distance	2	1541.726	376.753	0.000
Experimental Condition * Target Distance	8	1541.726	1.178	0.309

2.2.1.1.2 Pairwise Comparisons

(I)	(J)	Mean Diff. (I-J)	Std. Error.	df	Sig.**
Vibration Condition					
BI 90	BI 25	-0.016	0.007	1541.726	0.228
	NO VIB	-0.010	0.007	1541.726	1.000
	TRI 25	-0.014	0.007	1541.726	0.454
	TRI 90	-0.033	0.007	1541.726	0.000
BI 25	NO VIB	0.006	0.007	1541.726	1.000
	TRI 25	0.002	0.007	1541.726	1.000
	TRI 90	-0.016	0.007	1541.726	0.238
NO VIB	TRI 25	-0.004	0.007	1541.726	1.000
	TRI 90	-0.022	0.007	1541.726	0.018
TRI 25	TRI 90	-0.018	0.007	1541.726	0.112

(I)		(J)	Mean Diff. (I-J)	Std. Error.	df	Sig.**
Target Distance						
20%		40%	-0.118	0.006	1541.726	0.000
		80%	-0.142	0.006	1541.726	0.000
40%		80%	-0.024	0.006	1541.726	0.000

2.2.1.2 Stroke Patients

2.2.1.2.1 Tests of Fixed Main and Interaction Effects

Effect	Numerator df	Denominator df	F	Sig.
Intercept	1	4.991	267.000	0.000
Vibration condition	4	768.710	16.673	0.000
Target Distance	2	768.718	36.477	0.000
Experimental Condition * Target Distance	8	768.705	2.096	0.034

2.2.1.2.2 Pairwise Comparisons

(I)		(J)	Mean Diff. (I-J)	Std. Error.	df	Sig.**
Vibration Condition						
BI 90		BI 25	-0.029	0.013	769.264	0.238
		NO VIB	-0.047	0.013	768.348	0.002
		TRI 25	-0.072	0.013	768.348	0.000
		TRI 90	-0.094	0.013	768.348	0.000
BI 25		NO VIB	-0.018	0.013	769.264	1.000
		TRI 25	-0.043	0.013	769.264	0.007
		TRI 90	-0.065	0.013	769.264	0.000
NO VIB		TRI 25	-0.026	0.013	768.348	0.445
		TRI 90	-0.047	0.013	768.348	0.002
TRI 25		TRI 90	-0.022	0.013	768.348	0.892
Target Distance						
20%		40%	-0.052	0.010	768.903	0.000
		80%	-0.083	0.010	768.903	0.000
40%		80%	-0.031	0.010	768.348	0.006
Vibration Condition * Target Distance						
20%	BI 90	BI 25	-0.030	0.022	770.965	1.000

		NO VIB	-0.052	0.022	768.348	0.177
		TRI 25	-0.093	0.022	768.348	0.000
		TRI 90	-0.149	0.022	768.348	0.000
20%	BI 25	NO VIB	-0.022	0.022	770.965	1.000
		TRI 25	-0.062	0.022	770.965	0.049
		TRI 90	-0.119	0.022	770.965	0.000
20%	NO VIB	TRI 25	-0.041	0.022	768.348	0.656
		TRI 90	-0.097	0.022	768.348	0.000
20%	TRI 25	TRI 90	-0.056	0.022	768.348	0.104
40%	BI 90	BI 25	-0.038	0.022	768.348	0.804
		NO VIB	-0.067	0.022	768.348	0.023
		TRI 25	-0.087	0.022	768.348	0.001
		TRI 90	-0.085	0.022	768.348	0.001
40%	BI 25	NO VIB	-0.029	0.022	768.348	1.000
		TRI 25	-0.049	0.022	768.348	0.274
		TRI 90	-0.047	0.022	768.348	0.337
40%	NO VIB	TRI 25	-0.020	0.022	768.348	1.000
		TRI 90	-0.018	0.022	768.348	1.000
40%	TRI 25	TRI 90	0.002	0.022	768.348	1.000
80%	BI 90	BI 25	-0.017	0.022	768.348	1.000
		NO VIB	-0.021	0.022	768.348	1.000
		TRI 25	-0.037	0.022	768.348	0.914
		TRI 90	-0.047	0.022	768.348	0.317
80%	BI 25	NO VIB	-0.003	0.022	768.348	1.000
		TRI 25	-0.020	0.022	768.348	1.000
		TRI 90	-0.030	0.022	768.348	1.000
80%	NO VIB	TRI 25	-0.016	0.022	768.348	1.000
		TRI 90	-0.026	0.022	768.348	1.000
80%	TRI 25	TRI 90	-0.010	0.022	768.348	1.000

2.2.2 The Normalized Movement Time

2.2.2.1 Able-Bodied

2.2.2.1.1 Tests of Fixed Main and Interaction Effects

Effect	Numerator df	Denominator df	F	Sig.
Intercept	1	10.886	2.652	0.132
Vibration condition	4	1576.021	1.762	0.134
Target Distance	2	1576.021	3.962	0.019
Experimental Condition * Target Distance	8	1576.021	1.201	0.295

2.2.2.1.2 Pairwise Comparisons

(I)	(J)	Mean Diff. (I-J)	Std. Error.	df	Sig.**
Target Distance					
20%	40%	2.837	1.373	1576.021	0.117
	80%	3.691	1.373	1576.021	0.022
40%	80%	0.854	1.373	1576.021	1.000

2.2.2.2 Stroke Patients

2.2.2.2.1 Tests of Fixed Main and Interaction Effects

Effect	Numerator df	Denominator df	F	Sig.
Intercept	1	4.988	0.181	0.688
Vibration condition	4	707.375	7.525	0.000
Target Distance	2	707.385	12.473	0.000
Experimental Condition * Target Distance	8	707.367	1.978	0.047

2.2.2.2.2 Pairwise Comparisons

(I)	(J)	Mean Diff. (I-J)	Std. Error.	df	Sig.**
Vibration Condition					
BI 90	BI 25	5.169	3.062	708.598	0.918
	NO VIB	10.142	3.055	706.571	0.009
	TRI 25	14.298	3.055	706.571	0.000
	TRI 90	13.070	3.055	706.571	0.000
BI 25	NO VIB	4.973	3.062	708.598	1.000

		TRI 25	9.129	3.062	708.598	0.030
		TRI 90	7.901	3.062	708.598	0.101
NO VIB		TRI 25	4.157	3.055	706.571	1.000
		TRI 90	2.928	3.055	706.571	1.000
TRI 25		TRI 90	-1.228	3.055	706.571	1.000
Target Distance						
20%		40%	-2.594	2.369	707.793	0.822
		80%	-11.291	2.369	707.793	0.000
40%		80%	-8.697	2.366	706.571	0.001
Vibration Condition * Target Distance						
20%	BI 90	BI 25	0.124	5.326	712.517	1.000
		NO VIB	3.294	5.291	706.571	1.000
		TRI 25	12.553	5.291	706.571	0.179
		TRI 90	8.836	5.291	706.571	0.954
20%	BI 25	NO VIB	3.170	5.326	712.517	1.000
		TRI 25	12.428	5.326	712.517	0.199
		TRI 90	8.712	5.326	712.517	1.000
20%	NO VIB	TRI 25	9.258	5.291	706.571	0.806
		TRI 90	5.542	5.291	706.571	1.000
20%	TRI 25	TRI 90	-3.717	5.291	706.571	1.000
40%	BI 90	BI 25	0.449	5.291	706.571	1.000
		NO VIB	1.116	5.291	706.571	1.000
		TRI 25	4.960	5.291	706.571	1.000
		TRI 90	6.873	5.291	706.571	1.000
40%	BI 25	NO VIB	0.667	5.291	706.571	1.000
		TRI 25	4.511	5.291	706.571	1.000
		TRI 90	6.424	5.291	706.571	1.000
40%	NO VIB	TRI 25	3.844	5.291	706.571	1.000
		TRI 90	5.757	5.291	706.571	1.000
40%	TRI 25	TRI 90	1.913	5.291	706.571	1.000
80%	BI 90	BI 25	14.934	5.291	706.571	0.049
		NO VIB	26.015	5.291	706.571	0.000
		TRI 25	25.382	5.291	706.571	0.000
		TRI 90	23.501	5.291	706.571	0.000

80%	BI 25	NO VIB	11.081	5.291	706.571	0.366
		TRI 25	10.448	5.291	706.571	0.487
		TRI 90	8.567	5.291	706.571	1.000
80%	NO VIB	TRI 25	-0.633	5.291	706.571	1.000
		TRI 90	-2.514	5.291	706.571	1.000
80%	TRI 25	TRI 90	-1.881	5.291	706.571	1.000

2.2.3 The Ratio Between the Effective and Prescribed Index of Difficulty (IDe/ID)

2.2.3.1 Able-Bodied

2.2.3.1.1 Tests of Fixed Main and Interaction Effects

Effect	Numerator df	Denominator df	F	Sig.
Intercept	1	11.000	881.032	0.000
Vibration condition	4	514	1.322	0.261
Target Distance	2	514	120.656	0.000
Experimental Condition * Target Distance	8	514	0.717	0.677

2.2.3.1.2 Pairwise Comparisons

(I)	(J)	Mean Diff. (I-J)	Std. Error.	df	Sig.**
Target Distance					
20%	40%	-0.413	0.069	514.000	0.000
	80%	-1.068	0.069	514.000	0.000
40%	80%	-0.655	0.069	514.000	0.000

2.2.3.2 Stroke Patients

2.2.3.2.1 Tests of Fixed Main and Interaction Effects

Effect	Numerator df	Denominator df	F	Sig.
Intercept	1	5.000	520.325	0.000
Vibration condition	4	250.000	1.420	0.228
Target Distance	2	250	4.036	0.019
Experimental Condition * Target Distance	8	250	1.191	0.305

2.2.3.2.2 Pairwise Comparisons

(I)	(J)	Mean Diff. (I-J)	Std. Error.	df	Sig.**
Target Distance					
20%	40%	0.103	0.060	250.000	0.260
	80%	0.169	0.060	250.000	0.016
40%	80%	0.066	0.060	250.000	0.819
